# Link between carrot leaf secondary metabolites and resistance to *Alternaria dauci*

**DOI:** 10.1038/s41598-018-31700-2

**Published:** 2018-09-13

**Authors:** Claude Koutouan, Valérie Le Clerc, Raymonde Baltenweck, Patricia Claudel, David Halter, Philippe Hugueney, Latifa Hamama, Anita Suel, Sébastien Huet, Marie-Hélène Bouvet Merlet, Mathilde Briard

**Affiliations:** 1 IRHS, Université d’Angers, Agrocampus-Ouest, INRA, SFR 4207 QuaSaV, 49071 Beaucouzé, France; 2 SVQV, Université de Strasbourg, INRA, 68000 Colmar, France

## Abstract

Alternaria Leaf Blight (ALB), caused by the fungus *Alternaria dauci*, is the most damaging foliar disease affecting carrots (*Daucus carota*). In order to identify compounds potentially linked to the resistance to *A. dauci*, we have used a combination of targeted and non-targeted metabolomics to compare the leaf metabolome of four carrot genotypes with different resistance levels. Targeted analyses were focused on terpene volatiles, while total leaf methanolic extracts were subjected to non-targeted analyses using liquid chromatography couple to high-resolution mass spectrometry. Differences in the accumulation of major metabolites were highlighted among genotypes and some of these metabolites were identified as potentially involved in resistance or susceptibility. A bulk segregant analysis on F3 progenies obtained from a cross between one of the resistant genotypes and a susceptible one, confirmed or refuted the hypothesis that the metabolites differentially accumulated by these two parents could be linked to resistance.

## Introduction

Plants produce a large number of secondary metabolites that are involved in different aspects of plant life, including environmental adaptation, pollinator attraction, plant-plant interaction, scent, and defense against pathogens and herbivores^1–4^. Metabolomic approaches have been successfully used for the analysis of such metabolites involved in plant-pathogen interaction. To name just a few, Sade *et al*.^5^ used LC-MS and GC-MS analyses to provide a general overview of the metabolome changes upon infection of resistant and susceptible tomato cultivars by the tomato yellow leaf curl virus. Cuperlovic-Culf *et al*.^6^ assessed metabolic changes in spikelets of wheat varieties following *Fusarium graminearum* infection providing metabolic markers discriminating resistance level in wheat subtypes. While targeted metabolomic analysis measures the concentrations of a predefined set of metabolites, non-targeted or global metabolomic analysis allows for an assessment of the metabolites extracted from a sample, revealing a wide range of metabolite classes. Unfortunately, by this way, not all metabolite classes are obtained simultaneously, as many factors affect metabolite recovery and a large number of unknown metabolites remains unannotated in metabolite databases^7^. Using both targeted and non-targeted approaches allows a powerful investigation into mechanisms of metabolic variations in plants^8^.

Numerous studies have assessed the antimicrobial and antifungal activities of some secondary metabolites including volatile compounds (terpenes) and flavonoids in different pathosystems^9–13^. As reported by Singh and Sharma^14^, terpenes may act as phytoalexines as found in the interaction between *Oryza sativa* and *Magnaporthe grisae* offering antimicrobial properties^15^. Rodriguez *et al*.^16^ highlighted that while the up-regulation of some terpenoids is generally associated with plant defense properties, the down regulation of their production may sometimes reduce the susceptibility to specific pests or microorganisms. Flavonoids are also involved in plant-defense mechanisms, e.g. as cell-wall components, they seem to be an important part of the early barrier against fungal pathogens. More accumulated on the walls of the resistant varieties of cotton than in the suceptible ones, they are involved in the containment of *Verticillium dalhiae*^17^. Dehydrodimers of ferulic acid content in pericarp and aleurone tissues may play a role in resistance of maize to *Fusarium graminearum*^18^.

Up to now, no major resistance gene against Alternaria leaf blight (ALB), the most damaging foliar disease affecting this crop^19^, has been identified, all existing resistant cultivars are only partially resistant and fungicide treatments are still needed. One of the main breeding objectives of carrot seed companies is thus to increase the level of resistance of new cultivars by accumulating complementary resistance factors in one genotype. For this purpose, more than 300 accessions from our carrot genetic resources in Angers (France) and other European genetic resources were screened for their resistance to *Alternaria dauci* in different environments and over several years between 1997 and 2000. Some of these accessions were used to initiate a breeding program. Based on these tests, three inbred lines were selected to study the genetic determinism of carrot resistance to *A. dauci*, i.e. K3 and I2, highly resistant and H1, highly susceptible to the pathogen^12^. Different alleles for several quantitative trait loci (QTLs) associated with variations in the level of resistance were identified from these different genotypes suggesting different resistance mechanisms underlying these QTLs^20,21^. From these results, the goal for breeders is to obtain cultivars with improved resistance as, for example, the variety Boléro, which is one of the most resistant variety to *A. dauci*. This could probably be done by cumulating different favorable mechanisms. However, to effectively guide breeding programs, these mechanisms underlying these QTLs still need to be deciphered.

In collaboration with pathologists, we showed that two carrot secondary metabolites, falcarindiol and 6-methoxymellein, inhibited the development of *A. dauci* conidia *in vitro*. Differences in the level of falcarindiol accumulation in the leaves of resistant and susceptible cultivars were also identified, suggesting that this secondary metabolite could play a role in the resistance against *A. dauci*^22^. In addition, preliminary analyses on secondary leaf metabolism of a small set of genotypes in response to *A. dauci* revealed major differences in the profiles of secondary metabolite families. Particularly the levels of accumulation of some volatile compounds and hydroxycinammic acids were different not only between resistant and susceptible plant genotypes but also among resistant ones. Indeed, the total amount of monoterpenes after inoculation (in mg/kg of Internal Standard Equivalent) was significantly higher in K3 (1553 mg/kg) than in the suceptible genotype H1 (657 mg/kg) and the two resistant gentoypes, Bolero (537 mg/kg) and I2 (543 mg/kg). Similar conclusions were obtained for the total amount of sesquiterpenes. In addition, the total amount of hydroxycinammic acids (in mg/100 g ISE) was higher in K3 (102) than in H1 (72), Bolero (71) and I2 (44). These results confirmed metabolic differences between the different sources of resistance, which strengthened hopes of accumulating complementary mechanisms to obtain higher resistance and prompted us to investigate carrot secondary metabolism further.

Our goal in the present study was to investigate carrot secondary metabolism to identify compounds potentially influencing the interaction between the plant and the fungus *Alternaria dauci*. For this purpose, we chose two approaches: a targeted analysis focused on terpenes, and a non-targeted analysis of methanolic leaf extracts. The three carrot genotypes described above (Bolero, I2 and K3) and probably harbouring different mechanims of resistance to *A. dauci* and one highly susceptible genotype were evaluated in three consecutive years in three different environments, after inoculation or natural infestation.

Environments were chosen in order to span a vast array of conditions in terms of climate (controlled conditions under tunnel or field conditions), soil (sandy-clay or black sandy soils), latitude (North or South of France) and pathogen diversity, i.e. one reference *A. dauci* strain P2 (FRA018)^15^ under tunnel versus a large diversity of the fungus in the field^23^. Comparing the carrot genotypes in such different environmental conditions has allowed to focus on robust metabolic traits. By this way, we investigated not only the diversity of secondary metabolites among genotypes but also their stability across years and environments. The putative role of different metabolites in the resistance to *A. dauci* is discussed.

## Materials and Methods

### Plant materials

Three different carrot lines (H1, I2 and K3) and one cultivar (Boléro) were used for this experiment. Of the three lines, I2 and K3 are partially resistant to *A. dauci* with a rather high level of resistance while H1 is susceptible, as detailed in Le Clerc *et al*.^21^. I2 and K3 are two S2 lines of Asian origin developed by the Agrocampus-Ouest Institute, France. H1 is a S3 line of French origin produced by the HMClause breeding program. Boléro is a commercial hybrid from Vilmorin and is considered as the reference resistance against *A. dauci* for cultivated carrots. The resistance scores of the four genotypes in numerous environments over a period of 10 years (from 2006 to 2016) using the method described by Le Clerc *et al*.^20^ based on a 0 to 9 scale (0 = no visible disease damage on leaves, 9 = leaves totally blighted), ranked the genotypes as follows: K3 the most resistant with an average score of 3.4 followed by I2 (3.8), Boléro (4.3) and H1 (7)) (Supplementary Table S1). Two sets of bulk genotypes, ten resistant and ten susceptible, were also used for the experiment. These genotypes were F3 progenies obtained from a cross between the susceptible line H1 and the resistant line K3. They were selected according to their resistance score against *A. dauci* across three years of field phenotyping in Les Landes, France (Supplementary Table S1).

In the present paper, the term “genotype” is used to refer to lines, cultivars and F3 progenies.

### Culture conditions

The study was carried out over three years in three different environments. In 2014, only Boléro, H1, I2 and K3 were evaluated under tunnel conditions in Angers. This environment is our reference for all our research trials, while the field trials are performed in Les Landes and Gironde, the reference area of french carrot production (45% of the national tonnage). In 2015 and 2016, all 24 genotypes were grown in field conditions at Blagon (Latitude 44.7835; Longitude −0.9319, Gironde, France) and Ychoux (Latitude 44.3333; longitude −0.9667 Les Landes, France), respectively. Both are carrot production areas where *A. dauci* infection occurs naturally. For each trial, about 180 seeds per genotype were sown in an area of two meters in randomized blocks with two repetitions per genotype except for lines and cultivars, which were repeated three and four times in 2014 and 2016 respectively. Each field trial was conducted with local production conditions used by the producers.

In 2014, the tunnel experiment was performed in Angers (Latitude 47.4711; Longitude −0.5518 Maine et Loire, France) as described by Pawelec *et al*.^24^. Seeds were sown in sandy-clay soil in week 23. The plants were inoculated with P2, a moderately aggressive *A. dauci* strain^25^. A double inoculation, the first at the four-leaf stage (week 29) and the second two weeks later was performed to ensure the attack was successful. The inoculum was a conidial suspension prepared as described by Pawelec *et al*.^24^.

In 2015 and 2016, seeds were sown in black sandy soils in weeks 31 and 25, respectively. In both field conditions, the first pathogen attacks were predicted with the Plant-Plus system® developed by Dacom (http://www.dacom.nl/). This software predicts the risk of attack risk according to the weather conditions, plant development stage and pathogen concentration in the carrot production area concerned.

Both in the tunnel and in the fields, *A. dauci* attacks were confirmed by scoring symptoms and sampling leaves. The disease was assessed in October during the symptomatic phase as described above using the 0 to 9 scale. For pathogen identification, small pieces of leaves showing symptoms were disinfected from bacteria in 1° sodium hypochlorite for one minute, rinsed in sterile water then dried on a sterile blotter. The leaves were then incubated on malt agar medium in Petri dishes at 22 °C for three days. All this work was carried out in a sterile environment. After the three days, *A. dauci* spores were observed under the microscope (Supplementary Fig. S1).

### Sampling design

Samples were harvested eight days after the second inoculation in 2014 (week 32) and eight days after the first important attack risk of *A. dauci* (Dacom index >200) in week 39 in 2015 and in week 36 in 2016. A set of eight whole plants per repetition from each genotype (H1, I2 and K3) and the cultivar Boléro were harvested manually and put in Kraft paper bags. To ensure the samples remained fresh, the Kraft bags were immediately stored under wet sheets. The samples were transported from the field to the laboratory in a refrigerated truck at 8 °C, which corresponded to the room temperature of the final destination. In the tunnel experiment, the samples were transported directly to the lab in cold boxes.

The day after harvest, two intermediate leaves per plant were bulked for each genotype per repetition. Young leaves were not sampled as their development was not complete at sampling date and old leaves showing symptoms of senescence were not sampled too. Each bulked sample was frozen and ground in a mortar with liquid nitrogen before being stored at −80 °C. About 1 g of each bulk powder was freeze dried and ground again with iron beads using a MM2 Retsch mixer-mill to obtain a fine powder suitable for analyses.

### Targeted analyses- SPME-HS-GC-MS

Vials with 20 mL headspace (HS) each containing 25 mg of fresh frozen roughly ground carrot leaves were filled with 2 ml of Na_2_SO_3_ solution (10 g/L) and 3-octanol (50 µg) was added as internal standard. Each sample was incubated for 15 min at 35 °C. The volatile compounds were extracted under agitation (1000 g) with a divinylbenzene/Carboxen/polydimethylsiloxane fiber (1 cm, 23-gauge, 50/30 µm DVB/CAR/PDMS, Supelco, Bellefonte, PA) at 35 °C for 15 min fitted to a Gerstel MPS2 autosampler. The GC (Agilent 6890 Gas Chromatograph) was fitted with a DB-Wax column (i.d.: 30 m × 0.32 mm, film thickness: 0.5 µm). Helium was used as carrier gas with a column flow rate of 1.3 mL min^–1^. Volatiles were desorbed from the fiber in the GC inlet (220 °C) for 3 min and separated using the following temperature program: 40 °C for 5 min, increasing by 3 °C/min to 240 °C, then held for 5 min. The MS (Agilent 5973 N Mass Spectrometer) transfer line and ion source temperatures were set at 270 °C and 230 °C, respectively. The MS was operated in electron ionization mode and positive ions at 70 eV were recorded with a scan range from *m*/*z* 30 to *m*/*z* 300. ChemStation software (G1701DA, Rev D.03.00) was used for instrument control and data processing. The identity of the detected volatiles was determined by comparing their mass spectra with those of authentic standards and spectral libraries. The U.S. National Institute of Standards and Technology (NIST-05a), and the Wiley Registry 7th Edition mass spectral libraries were used for identification. Match thresholds of spectra were at least 85% and retention index were taken into account for compound identification. Data are presented as normalized peak area per mg of fresh weight.

For targeted analyses using UHPLC-ESI-MS, the exact *m/z* and retention time of each selected metabolite were used for relative quantification using the Excalibur software and the integration of each peak was checked manually before validation.

### Non-targeted analyses- UHPLC-ESI-MS

LC-MS grade methanol and acetonitrile were purchased from Roth Sochiel (Lauterbourg, France), water was provided by a Millipore water purification system. Chlorogenic acid, luteolin 7*-O-*rutinoside and luteolin 7*-O-*glucuronide were purchased from Sigma-Aldrich (Saint-Quentin Fallavier, France). apigenin 7*-O-*glucoside, luteolin 4′*-O-*glucoside and luteolin 7*-O-*glucoside were purchased from Extrasynthese (Lyon, France).

About 10 mg of fine freeze-dried leaf powder were extracted with 150 µL/mg of methanol containing 5 µg/mL of phenyl glucoside as internal standard. The extract was then subjected to a quick vortex before being placed in an ultrasound bath for 10 minutes. A heating step at 60 °C for 30 minutes in a water bath was followed by centrifugation at 13000 g at 4 °C for 10 minutes. Supernatant (150 µL) was collected in a vial. Metabolites were analyzed using a UHPLC system (Dionex Ultimate 3000; Thermo Fisher Scientific) equipped with a diode array detector (DAD). Chromatographic separation was performed on a Nucleodur HTec column (150 × 2 mm, 1.8 µm particle size; Macherey-Nagel) maintained at 30 °C. The mobile phase consisted of acetonitrile/formic acid (0.1%, v/v) (eluant A) and water/formic acid (0.1%, v/v) (eluant B) at a flow rate of 0.25 ml/ min. The gradient elution program was as follows: 0–4 min, 80–70% B; 4–5 min, 70–50% B; 5–6.5 min, 50% B; 6.5–8.5 min 50–0% B; 8.5–10 min 0% B. The injected volume of sample was 1 µL. The liquid chromatography system was coupled to an Exactive Orbitrap mass spectrometer (Thermo Fisher Scientific) equipped with an electrospray ionization source operating in positive mode. The instruments were controlled with Xcalibur software (Thermo Fischer). The ion transfer capillary temperature was set at 300 °C and the needle voltage at 3400 V. Nebulization with nitrogen sheath gas and auxiliary gas were maintained at 40 and 5 arbitrary units, respectively. The spectra were acquired within the mass-to-charge ratio (*m/z*) range of 95–1200 atomic mass units (a.m.u.), using a resolution of 50 000 at *m/z* 200 a.m.u. The system was calibrated internally using dibutyl phthalate as lock mass (*m/z* 279.1591), giving a mass accuracy <1 ppm.

The raw data from each line and cultivar sample and from all three years (32 samples) were converted into mzXML format using MSConvert. mzXML data were sorted into four classes according to genotype and then processed using the XCMS software package^26^. Settings of the xcmsSet function of XCMS were as follows: the method to extract and detect ions used was “centWave”, ppm = 2, noise = 30 000, mzdiff = 0.001, prefilter = c (5.15000), snthresh = 6, peak width = c (6.35). Peaks were aligned using the obiwarp function using the following group density settings: bw = 10, mzwid = 0.0025, minimum fraction of samples for group validation: 0.5. Ion identifiers were generated by XCMS script as MxxxTyyy, where xxx is the *m/z* and yyy the retention time in seconds.

### Data output and statistical analyses

For all the chemical analyses, using the raw data of the lines and cultivar together with all the replicates and after checking for residual normality and variance homogeneity, we analyzed the environmental and genotype effects on the accumulation of secondary metabolites using analysis of variance. When these postulates were not verified, a Kruskal-Wallis test was used. Then, to focus on the effect of genotype, the accumulation value of each secondary metabolite from each genotype replicate (H1, I2, K3 and Boléro) per year was autoscaled i.e. centered-scaled, as described by van den Berg *et al*.^27^, according to the mean and standard deviation of the accumulation of the metabolite concerned per year. As explained by the authors, as large differences in concentration for different metabolites in a metabolomics data set are not proportional to the biological relevance of these metabolites, autoscaling can be used, as it is able to remove the dependence of the rank of the metabolites on the average concentration and the magnitude of the fold changes. All metabolite become equally important. As for one given metabolite, large variations in concentration may be due to different environmental conditions between years or experimental trials, autoscaling limits these environmental effects in order to highlight genotype-dependent differences. From these autoscaled values, a principal component analysis (PCA) was performed of each sub-family of metabolites per year or across the three years. In addition, an analysis of variance (ANOVA) followed by a Tukey’s HSD test was performed to identify the most discriminant metabolites in each sub-family and to distinguish between susceptible (H1) and resistant genotypes (I2, K3 and Boléro). The accumulation of secondary metabolites differentially accumulated in H1 and K3 was investigated in resistant and susceptible bulk sets of F3 progenies with a Student’s t-test. All statistical analyses were generated with RStudio team version 1.0.136 (2016).

## Results

### Environmental and genotype effect on secondary metabolite accumulation in carrot leaves

Three highly resistant genotypes representing different geographical origin and genetic background and one highly susceptible genotype were studied in four different environments to identify the origin of the metabolic variation. Variance analysis of raw data showed that environmental effects influenced the accumulation of all sub-families of secondary metabolites (Table 1). Sesquiterpenes and flavonoids varied up to 10 fold between environments, while monoterpenes were less influenced by environmental conditions. Only sesquiterpenes showed an environment x genotype interaction, however it was very low compared with the environmental or genotype effects. This low interaction enabled subsequent genotype analyses in all environmental conditions. Autoscaling data were able to effectively eliminate environmental effects to make it possible to focus on genotype effects (last column in Table 1: no significant P Value for environment).

**Table 1:** Environmental and genotype effects on the accumulation of each sub-family of identified secondary metabolites in carrot leaves, using analysis of variance. Pr (>F) significance codes: <0.001 ‘***’ 0.001 ‘**’ 0.01 ‘*’ 0.05 ‘.’ ^a^Kruskal-Wallis test on raw data; ^b^Anova based on autoscaled values.

Sub-Family	Factor	Pr(>F)^a^	Pr(>F)^b^
monoterpenes	Environment	0.0488*	1.000
	Genotype	0.4975	0.363
	Genotype:Environment	0.1236	0.435
sesquiterpenes	Environment	2.287e-08***	1.000
	Genotype	1.471e-07***	1.82e-05***
	Genotype:Environment	0.0308*	0.318
chlorogenic acids	Environment	0.00783**	0.998
	Genotype	0.36994	0.000812***
	Genotype:Environment	0.89289	0.423
flavones	Environment	0.0026**	0.992
	Genotype	0.1232	0.0622
	Genotype:Environment	0.2344	0.6667

### Analysis of terpene volatiles in carrot leaf extracts

SPME-GC-MS analysis of carrot leaves was performed to identify and relatively quantify terpenes of the four genotypes. It resulted in the identification and relative quantification of 30 terpenes, including 15 monoterpenes and 15 sesquiterpenes (Table 2; Supplementary Tables S2 and S3). In all genotypes, whatever the environment and based on raw data, the main monoterpenes were β-myrcene, sabinene, α-pinene and limonene, and the main sesquiterpenes were caryophyllene and germacrene D.

**Table 2:** List of 15 monoterpenes and 15 sesquiterpenes identified by SPME-GC-MS in the carrot leaves of all genotypes (H1, Boléro, I2 and K3) over three years (Angers 2014, Blagon 2015 and Ychoux 2016) the sub-families are ranked from those with the biggest to the smallest quantity.

Monoterpenes	Sesquiterpenes
β-myrcene	caryophyllene
sabine	germacrene D
α-pinene	trans-α-farnesene
limonene	α-humulene
trans-β-ocimene	β-selinene
cis-β-ocimene	δ-cadinene
p-cymene	trans-α-bergamotene
γ-terpinene	α-bisabolene
β-phellandrene	cis-β-farnesene
α-terpinolene	α-amorphene
β-pinene	β-bisabolene
cis-rose oxide	β-cubebene
camphene	cis-α-bergamotene
bornyl acetate	α-copaene
linalol	trans-β-farnesene

**Figure 1:**
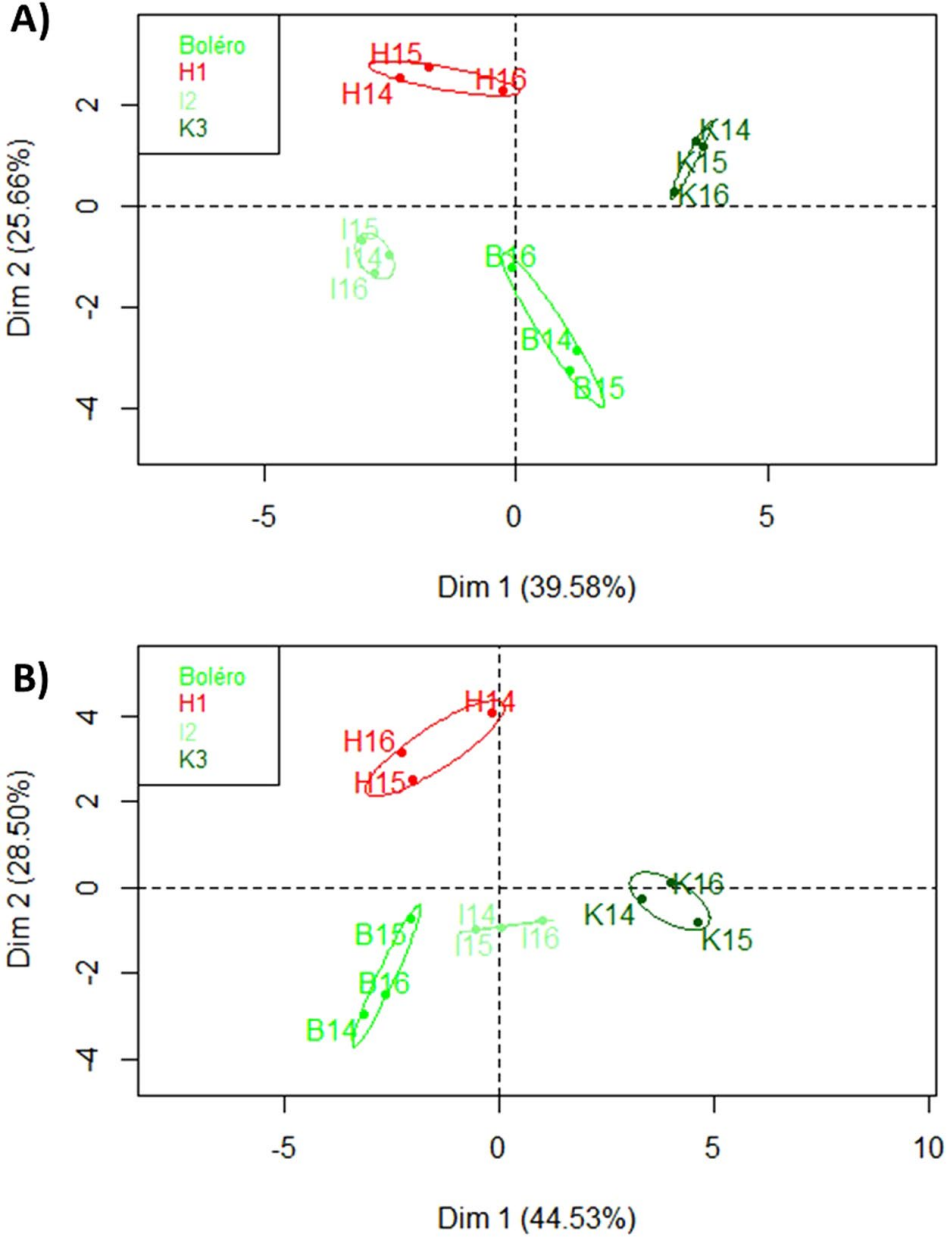
Principal component analysis (PCA) of terpene leaf volatiles in the selected carrot genotypes, (**A)** PCA of the accumulation of monoterpenes. (**B**) PCA of the accumulation of sesquiterpenes. Carrot leaves were analyzed by GC-MS across the three years and environmental conditions (2014 Angers (Tunnel), 2015 Blagon (Field) and 2016 Ychoux (Field)). Codes correspond to genotype followed by the year of the experiment, for example “H14” corresponds to genotype H1 grown in summer 2014 in Angers under tunnel conditions. Confidence ellipses are designed with the “plotellipse” function of the factominer package of Rstudio.

Based on the autoscaled data of each genotype (Supplementary Table S4), we next performed a principal component analysis (PCA). The individual map of the PCA (Fig. 1) shows that individuals clustered according to genotype whatever the environment except for slight differences concerning monoterpenes in Boléro_16, and sesquiterpenes in H1_14 and Boléro_15. The PCA also highlighted differences between the four genotypes (H1, I2, K3 and Boléro).

ANOVA identified 10 monoterpenes and 11 sesquiterpenes, enabling us to distinguish between the four genotypes. Seven metabolites (Table 3) γ-terpinene, camphene, limonene, α-pinene (monoterpenes) and cis-α-bergamotene, trans-β-farnesene and cis-β-farnesene (sesquiterpenes) differentiated H1 from all three resistant (I2, K3 and Boléro) genotypes. Among the 15 terpene metabolites differentially accumulated by K3 in comparison to H1, in addition to the above seven metabolites common to the three pairs, two of them were also differentially accumulated by I2 and one by Boléro. Finally, one metabolite was common to the H1-I2 and H1-Boléro pairs (Table 3). The discrimination among resistant genotypes already highlighted in Fig. 1 is consistent with the accumulation of 11, 13 and 8 terpene metabolites for K3-I2, K3-Boléro and I2- Boléro pairs, respectively (Table 3).

**Table 3:** List of terpenes analyzed across the three years and environmental conditions (auto-scaled values) differentiated between genotypes (H1, Boléro, I2 and K3) by ANOVA with a p value < 0.025 and Tukey’s honest significant difference (HSD) test, which highlighted pairs of genotypes with significant differences in accumulation of each metabolite (x).

Metabolite		P value	Tukey’s (HSD) between H1 and resistant genotypes			Tukey’s (HSD) among resistant genotypes		
			H1-K3	H1-I2	H1-Boléro	K3-I2	K3-Boléro	I2-Boléro
Mono-terpenes	γ-terpinene	1.14E-05	x	x	x	x	x	x
	camphene	0.000568	x	x	x			x
	limonene	0.000832	x	x	x			
	α-pinene	0.0226	x	x	x			
	cis-β-ocimene	8.84E-09	x	x		x	x	
	α-terpinolene	0.00518		x	x			
	bornyl acetate	9.84E-07	x			x	x	
	sabinene	0.00123		x		x	x	
	p-cymene	0.00518		x		x		x
	β-myrcene	0.0107	x			x		
Sesqui-terpenes	cis-α-bergamotene	<2e-16	x	x	x			
	trans-β-farnesene	<2e-16	x	x	x			
	cis-β-farnesene	5.66E-12	x	x	x		x	x
	α-copaene	2.69E-07	x		x	x	x	x
	α-bisabolene	0.000879	x	x			x	x
	β-bisabolene	5.01E-06	x			x	x	
	α-amorphene	9.29E-05			x	x	x	x
	α-humulene	0.000288	x			x	x	
	δ-cadinene	0.000527			x		x	x
	germacrene D	0.00176			x		x	x
	caryophyllene	0.00328	x			x	x	

Within each sub-family metabolites are ranked from those with significant differences between H1 and the three resistant genotypes to those with significant differences between H1 and two or only one resistant genotype.

### Non-targeted metabolomic analysis of carrot leaf extracts

**Figure 2:**
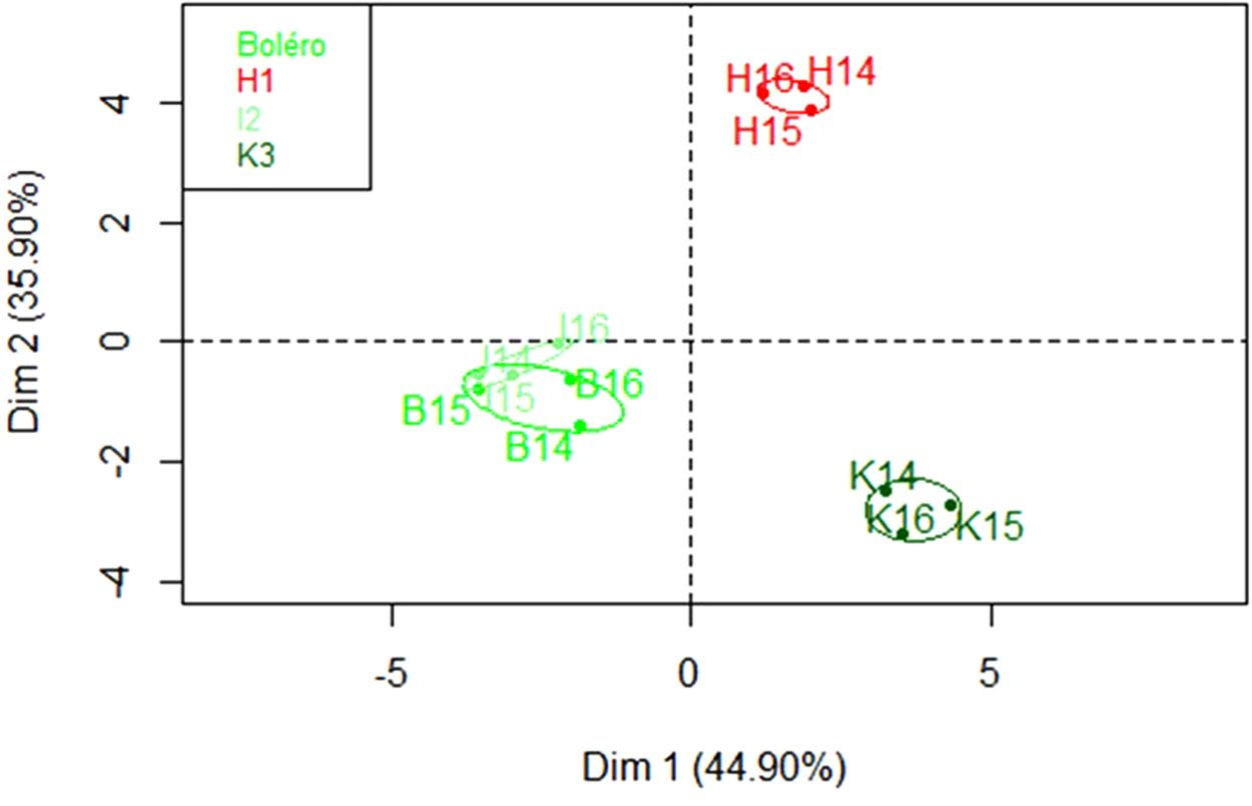
Principal component analysis of UHPLC-MS data. PCA was performed on a subset of 52 major ions (with peak area >2.10^6^, fold >2 or <0.5 and p-value < 0.01). Quantifications were performed on the samples collected over three years. Codes correspond to genotype followed by the year of the experiment, for example “H14” corresponds to genotype H1 grown in summer 2014 in Angers under tunnel conditions.

UHPLC-ESI-MS was used to profile the metabolites present in carrot leaves and to potentially identify new compounds. XCMS analysis resulted in a total of 754 ions for the three years and the four genotypes. Using analysis of variance, peak area and fold change, we selected 355 ions that differentiated the genotypes with a p-value < 0.01. Selection of major differential ions (peak area >2.10^6^, fold >2 or <0.5) between genotypes led to a final set of 52 major ions (Supplementary Tables S2 and S3; Supplementary Fig. S2). For metabolite identification, the Metlin database^28^ was searched for compounds possibly corresponding to the ions of interest (with 1 ppm average mass error). Submission of the 52 major ions to the Metlin database led to the putative identification of several compounds of interest. Based on retention time, the presence of fragments or isotopes related to specific pseudo-molecular ions was analyzed further. Among the selection of 52 major ions, 33 ions were attributed to 17 metabolites. Putative identifications were confirmed by comparing the retention times (RT) and mass spectra of selected metabolites to those of authentic standards (Fig. S3). PCA of these 52 ions led to a very clear separation of H1 and K3 from the less-resolved Boléro and I2 genotypes, the two PCA dimensions explaining a total of 69% of the variance (Fig. 2).

This set of 52 major ions (Supplementary Table S5) was then investigated further to characterize some of the associated metabolites. Submission of these 52 ions to the Metlin database^28^ led to the putative identification of several compounds of interest. Indeed, one of the proposed structures for compounds giving ion n°5 (M355T122) with the mass-to-charge ratio (*m/z*) 355.1023 was caffeoylquinic (chlorogenic) acid. Similarly, ion n°10 (M369T195) with *m/z* 369.11803 was potentially associated with feruloylquinic acid. Using the corresponding commercial standard, the identity of ion n°5 (M355T122) was confirmed as 3-caffeoylquinic acid (Fig. S3). Furthermore, some ions were of particular interest because their *m/z* 271, 287 and 301 may correspond to flavonoid structures. Based on their accurate *m/z*, the molecular formula C_15_H_11_O_5_ was attributed to the ion M271T307 (n°31), C_15_H_11_O_6_ to M287T268 (n°23) and C_16_H_13_O_6_ to M301T321 (n°41). These molecular formulas may correspond to flavone, isoflavone or flavanone structures. Flavones such as apigenin 7*-O-*glucoside and luteolin 7*-O-*glucoside have been previously reported in carrot leaves^29^. The ion with *m/z* 271.0599 may thus correspond to apigenin and the ion with *m/z* 287.0547, harboring one more oxygen molecule, may correspond to luteolin. Similarly, the molecular formula C_16_H_13_O_6_ attributed to the ion with *m/z* 301.0705 was consistent with the presence of a methyl group on luteolin. Methylated derivatives of luteolin such as chrysoeriol 7*-O-*glucoside have been reported in carrot leaves^30^. Indeed, extracted ion chromatograms (EIC) for *m/z* 271.0599, 287.0547 and 301.0705 indicate that several major compounds may share these flavone skeletons (Supplementary Fig. S2).

**Figure 3:**
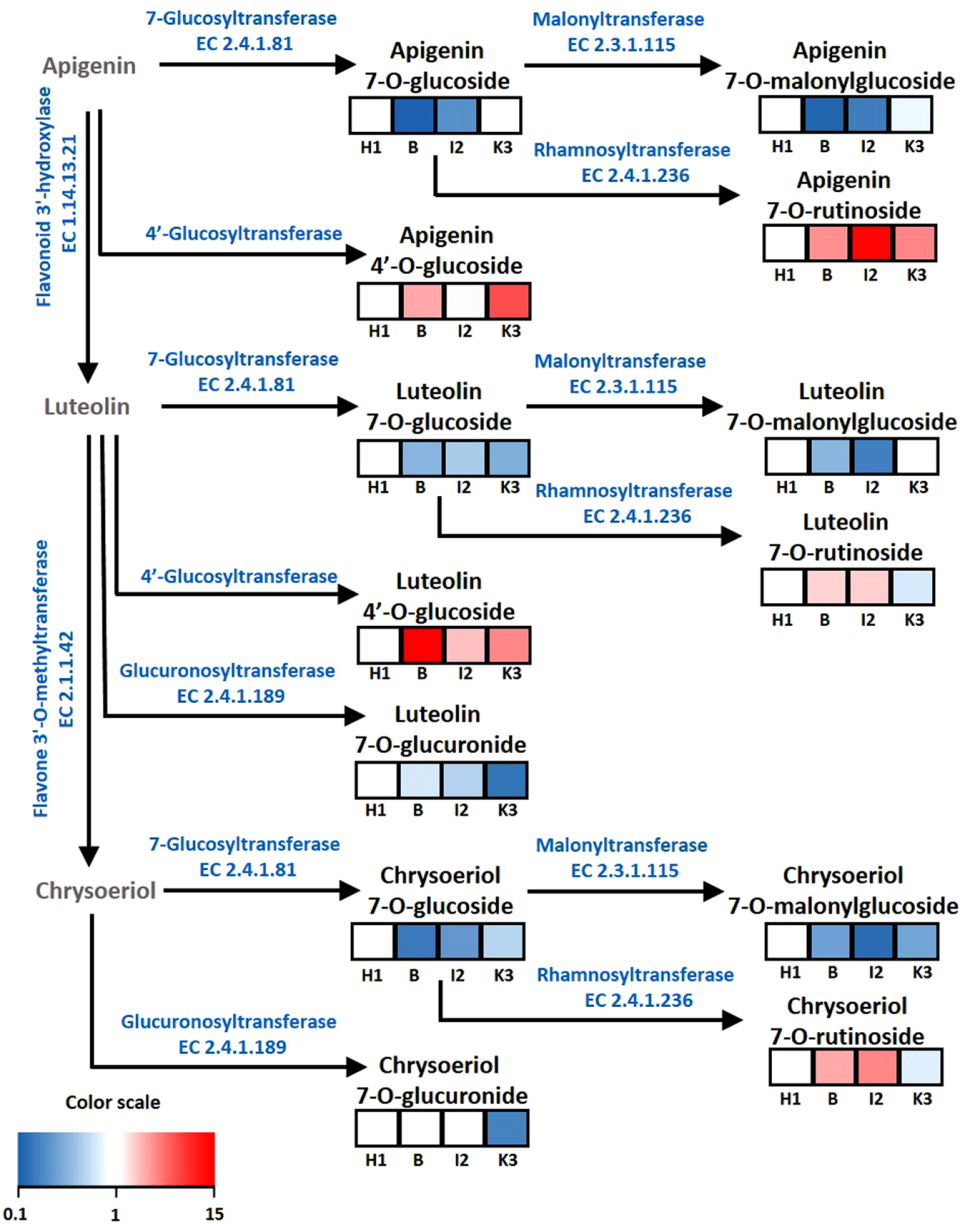
Biosynthetic pathway and patterns of accumulation of major flavonoids in different carrot genotypes. Indicated flavonoids were quantified in carrot leaf samples in a 3-year evaluation. Heat maps show fold changes in the accumulation of each flavonoid among the H1, Boléro, I2 and K3 genotypes, presented from left to right. Fold changes are indicated according to the scale bar and were calculated for each compound based on the content in the H1 genotype (auto-scaled values). The biosynthetic pathway was based on the KEGG « Flavone and flavonol biosynthesis » pathway (map00944, Kanehisa *et al*., 2017).

In both the carrot leaves extracts and the luteolin 7*-O-*glucoside commercial standard, in-source fragmentation produced a characteristic ion with *m/z* 287.0547 (C_15_H_11_O_6_), corresponding to the flavone backbone (Supplementary Table S6). EIC for *m/z* 287.0547 showed that several metabolites gave rise to this particular ion in carrot leaf extracts (Supplementary Fig. S2), indicating that these molecules may contain luteolin. A more detailed analysis of the mass spectra of some of these metabolites showed that they were actually present in our list of 52 major ions (Supplementary Table S5). This prompted us to characterize these putative luteolins containing compounds in more detail (Tables 4 and 5 and Supplementary Table S6). The mass and the formula of the compounds suggest they may correspond to glycoside derivatives, constituted by glucose, rutinose, glucuronide, or malonyl-glucose. By comparing their retention times and fragment patterns to those of authentic standards (Supplementary Table S6), the identity of M897T242 (n◦15), M449T312 (n°36), M463T272 (n°25) and M595T208 (n°11) was confirmed as luteolin 7*-O-*glucoside, luteolin 4′*-O-*glucoside, luteolin 7*-O-*rutinoside, and luteolin 7*-O-*glucuronide, respectively (Tables 4 and 5). Detailed analysis of the mass spectra of apigenin, luteolin and chrysoeriol derivatives (Supplementary Fig. S2 and Table S7) led to the putative identification of 13 flavonoids, five of which were confirmed using commercial standards (Tables 4 and 5). Together, these 13 compounds gave rise to 29 of the 52 major ions that best differentiated between the four genotypes. The structures of these 13 flavonoids are listed in Supplementary Table S7. In order to better visualize the relationships between the 13 flavonoids, they are positioned on a flavone biosynthetic pathway (KEGG map00944, Kanehisa *et al*.^31^) (Fig. 3). Quantification of these flavones in leaf samples of the four carrot genotypes, H1, I2, K3 and Boléro, revealed major differences in accumulation patterns, with fold changes ranging from two to 25 among genotypes. In particular, amounts of 4′*-O-*glucosylated flavones such as luteolin 4′*-O-*glucoside and putative apigenin 4′*-O-*glucoside were 10 to 25 times higher in the *A. dauci*-resistant Boléro, I2 and K3 genotypes than in the susceptible H1genotype.

**Table 4:** Flavonoids in carrot leaves that differentiated between genotypes.

Ion n°	Identifier	*m/z*	Formula	RT (min)	Identification
11	M595T208	595.1657	C_27_H_30_O_15_	3.46	**luteolin 7** ***-O-*** **rutinoside**
15	M897T242	449.1078	C_21_H_20_O_11_	4.05	**luteolin 7** ***-O-*** **glucoside**
21	M579T266	579.1708	C_27_H_30_O_14_	4.44	*apigenin 7-O-rutinoside*
25	M463T272	463.0872	C_21_H_18_O_12_	4.38	**luteolin 7** ***-O-*** **glucuronide**
27	M609T278	609.1812	C_28_H_32_O_15_	4.62	*chryoseriol 7-O-rutinoside*
35	M433T308	433.1128	C_21_H_20_O_10_	5.1	**apigenin 7** ***-O-*** **glucoside**
36	M449T312	449.1077	C_21_H_20_O_11_	5.19	**luteolin 4′** ***-O-*** **glucoside**
39	M433T320	433.1128	C_21_H_20_O_10_	5.3	*apigenin 4′-O-glucoside*
42	M463T322	463.1233	C_22_H_22_ O_11_	5.33	*chrysoeriol 7-O-glucoside*
44	M477T353	477.1028	C_22_H_20_O_12_	6.1	*chrysoeriol 7-O-glucuronide*
47	M519T387	519.1134	C_24_H_22_O_13_	6.4	*apigenin 7-O-malonylglc*
48	M535T389	535.1083	C_24_H_22_O_14_	5.33	*luteolin 7-O-malonylglc*
50	M549T391	549.1238	C_25_H_24_O_14_	6.5	*chryoseriol 7-O-malonylglc*

Identifications confirmed by using the corresponding standards are in bold, putative identifications are in *italics*. Ions are numbered according to their retention time. For all ions, the number, identifier, *m/z*, and retention time (RT) are indicated. All 52 ions selected by non-targeted metabolomics are listed in Supplementary Table S3.

**Table 5:** Putative and confirmed structures of the 13 flavonoids characterized in this work.


	R1	R2	R3
**luteolin 7** ***-O-*** **rutinoside**	**rutinosyl**	**H**	**OH**
**luteolin 7** ***-O-*** **glucoside**	**glucosyl**	**H**	**OH**
**luteolin 7** ***-O-*** **glucuronide**	**glucuronyl**	**H**	**OH**
**luteolin 4′** ***-O-*** **glucoside**	**H**	**Glucosyl**	**OH**
luteolin 7*-O-*malonylglc	malonyl-glucosyl	H	OH
apigenin 7*-O-*rutinoside	rutinosyl	H	H
**apigenin 7** ***-O-*** **glucoside**	**glucosyl**	**H**	**H**
apigenin 4′*-O-*glucoside	H	Glucosyl	H
apigenin 7*-O-*malonylglc	malonyl-glucosyl	H	H
chrysoeriol 7*-O-*rutinoside	rutinosyl	H	OCH_3_
chrysoeriol 7*-O-*glucoside	glucosyl	H	OCH_3_
chrysoeriol 7*-O-*malonylglc	malonyl-glucosyl	H	OCH_3_
chrysoeriol 7*-O-*glucuronide	glucuronyl	H	OCH_3_

Identifications confirmed by using the corresponding authentic standards are indicated in bold.

When focusing on the secondary metabolites that highly significantly differentiated between genotypes (Table 6), six flavonoid metabolites chrysoeriol 7*-O-*malonylglucoside, luteolin 4*-O-*glucoside, apigenin 7*-O-*rutinoside, apigenin 4′*-O-*glucoside, M611T366 and luteolin 7*-O-*glucoside were whown to differentiate H1 from all three resistant (I2, K3 and Boléro) genotypes. In addition to the six above listed metabolites common to the three pairs, two other flavonoid metabolites and 1 chlorogenic acid, were also differentially accumulated by K3 in comparison to H1 (Table 6). Finally, five metabolites were common to the H1-I2 and H1-Boléro pairs (Table 4).

**Table 6:** LC-MS secondary metabolites analyzed across the three years and environmental conditions (auto-scaled values) that differentiated between genotypes (H1, Boléro, I2 and K3) by ANOVA with a p value ≤ 0.01 and Tukey’s honest significant difference (HSD) test, which highlighted pairs of genotypes with significant differences in each metabolite accumulation (x).

Metabolite	P value	Tukey’s (HSD) between H1 and resistant genotypes			Tukey’s (HSD) among resistant genotypes		
		H1-K3	H1-I2	H1-Boléro	K3-I2	K3-Boléro	I2-Boléro
chrysoeriol 7*-O-*malonylglucoside	8.22E-07	x	x	x	x		x
luteolin 4′*-O-*glucoside	9.26E-07	x	x	x		x	x
apigenin 4′*-O-*glucoside	6.18E-06	x	x	x	x	x	x
M611T366	5.01E-05	x	x	x		x	
apigenin 7*-O-*rutinoside	4.00E-04	x	x	x	x		x
luteolin 7*-O-*glucoside	0.000455	x	x	x			
apigenin 7*-O-*malonylglucoside	3.38E-06		x	x	x	x	
luteolin 7*-O-*rutinoside	1.61E-05		x	x	x	x	
apigenin 7*-O-*glucoside	9.21E-05		x	x	x	x	
chrysoeriol 7*-O-*rutinoside	0.000191		x	x	x	x	
chrysoeriol 7*-O-*glucoside	0.00263		x	x		x	
feruloylquinic acid	5.69E-07	x			x	x	
luteolin 7*-O-*glucuronide	0.000206	x			x	x	
chrysoeriol 7*-O-*glucuronide	0.00199	x			x	x	

Metabolites are ranked from those with significant differences between H1 and the three resistant genotypes to those with significant differences between H1 and two or only one resistant genotype.

### Bulk segregant analysis of H1xK3 progenies

To confirm or refute the hypothesis that the metabolites differentially accumulated between H1 and K3 could be linked to resistance, we focused on metabolites that are differentially accumulated by H1 and K3. We analyzed a bulk of highly susceptible and a bulk of highly resistant progenies originated from the cross between these two lines.

As shown above, the accumulation of 24 metabolites (15 terpenes, 8 flavonoids and 1 chlorogenic acid) differed significantly between HI and K3 (Tables 3 and 6). Comparison of the two bulk sets of resistant and susceptible F3 progenies obtained from the cross between HI and K3 revealed highly significant statistical differences (P value < 0.01) in five of them. Luteolin 7*-O-*glucuronide was highly accumulated by susceptible progenies and by H1. Conversely, feruloylquinic acid, camphene, α-pinene and apigenin 4′*-O-*glucoside were more accumulated in resistant genotypes (Table 7). Three additional metabolites revealed significant statistical differences (P value ≤ 0.05): luteolin 4′-*O*-glucoside, caryophyllene and β-bisabolene. The other secondary metabolites showed no significant statistical difference (Table 7).

**Table 7:** Results of Student’s t-test comparing resistant and susceptible genotype bulks highlighting statistical difference for some secondary metabolites differentially accumulated by H1 and K3. SE = standard error.

Secondary metabolites	Mean (±SE)		P value
	Resistant	Susceptible	
luteolin 7*-O-*glucuronide	−0.701 (0.163)	0.701 (0.147)	1.755e-07
feruloylquinic acid	0.561 (0.235)	−0.561 (0.108)	1.811e-04
camphene	0.430 (0.230)	−0.430 (0.166)	0.004
α-pinene	0.418 (0.227)	−0.418 (0.173)	0.005
apigenin 4′*-O-*glucoside	0.400 (0.247)	−0.400 (0.149)	0.009
luteolin 4′*-O-*glucoside	0.319 (0.245)	−0.319 (0.170)	0.03
caryophyllene	0.301 (0.253)	−0.301 (0.163)	0.05
β-bisabolene	0.304 (0.278)	−0.304 (0.113)	0.05
α-bisabolene	0.252 (0.261)	−0.252 (0.158)	0.10
apigenin 7*-O-*rutinoside	0.226 (0.132)	−0.226 (0.278)	0.15
bornyl acetate	0.217 (0.277)	−0.217 (0.135)	0.16
cis-α-bergamotene	−0.115 (0.229)	0.115 (0.214)	0.46
cis-β-farnesene	−0.110 (0.233)	0.110 (0.210)	0.48
α-humulene	0.107 (0.182)	−0.107 (0.256)	0.50
cis-β-ocimene	−0.092 (0.167)	0.092 (0.266)	0.55
α-Coapene	−0.094 (0.237)	0.094 (0.206)	0.55
luteolin 7*-O-*glucoside	−0.068 (0.228)	0.068 (0.217)	0.66
trans-β-farnesene	−0.066 (0.222)	0.066 (0.224)	0.67
apigenin 7*-O-*malonylglucoside	−0.065 (0.158)	0.065 (0.273)	0.68
chrysoeriol 7*-O-*malonylglucoside	0.028 (0.192)	−0.028 (0.251)	0.85
β-myrcene	−0.026 (0.234)	0.026 (0.212)	0.86
limonene	−0.022 (0.232)	0.022 (0.215)	0.88
γ-terpinene	0.001 (0.197)	−0.001 (0.247)	0.99

Mean and SE are autoscaled values. P value ≤ 0.05 indicate statistical difference between resistant and susceptible genotypes. Secondary metabolites are ranked from the most to the least significant P value.

## Discussion

The main objective of this paper is to identify carrot leaf metabolites that could be good candidates to explain resistance mechanisms of carrot to *A. dauci*. The major terpene leaf compounds found in the present study (Table 2) are in accordance with results already reported in the literature: Kainulainen *et al*.^32^ reported myrcene, sabinene, trans-α-ocimene, limonene, germacrene D, and trans-α-caryophyllene as major leaf compounds of two carrot varieties. Ulrich *et al*.^33^ reported germacrene, β-caryophyllene, limonene, β-myrcene, sabinene and α-pinene as major compounds in 10 carrot cultivars, and finally, Keilwagen *et al*.^34^ reported β-myrcene, β-caryophyllene and limonene as the most abundant compounds in leaves of a panel of 85 carrot cultivars and accessions. Concerning flavonoids, a very significant work was conducted in the present study to provide a detailed description of the luteolin and apigenin derivatives biosynthetic pathway (Fig. 3), that was previously largely unclear in the literature. Better description of this pathway will pave the way for functional and genetic analysis of flavone biosynthesis in carrots.

As reviewed by Verma and Shukla^35^ numerous factors including ontogenic or morphogenetic factors may be responsible for fluctuations in plant secondary metabolites. Because in the present study samples were taken from the same plant organs at a similar development stage each year, i.e. the fourth and fifth leaves, these factors were assumed to have no influence.

The analyses of environment effects on the overall accumulation level of secondary metabolites per sub-family (chlorogenic acids, flavones, mono- and sesquiterpenes) revealed statistical differences between the three environments studied (Angers tunnel, Blagon field and Ychoux field; Table 1). This has already been described in a very high number of papers where biotic or abiotic environmental factors are cited as responsible for fluctuations in the accumulation of plant secondary metabolites, even for the carrot species^33,36–39^. In the present study, the overall accumulation level of secondary metabolites could also be explained by a combination of different biotic and abiotic factors, however we were not able to distinguish one from the other, and the main focus of the present paper is genetics.

While the accumulation level of secondary metabolites in one genotype could differ between environments, the ranking of genotypes was always the same whatever the year, leading to the absence of a genotype x environment interaction. The autoscaled values clearly showed that the carrot secondary metabolism was strongly affected by genotype while, in the raw data, this was masked by the environmental effect (Table 1). Some studies also reported a genotype effect. Kainulainen *et al*.^32^ compared the volatile emissions from the leaves of two different carrot cultivars grown in field or greenhouse conditions and found that the difference in volatile compounds was only due to a genotype effect. Ibrahim *et al*.^37^ demonstrated that, under high temperatures, the cv Parano accumulated less trans-β-ocimene, trans-α-bergamotene, trans-β-farnesene and more total phenolic compounds than the cv Splendid. As we chose the four genotypes based on their different level of resistance to *A. dauci* (Table S1), we hypothesized that the genotype effect revealed in the present study could be explained by the resistance status of one genotype. The resistance scores obtained in the tunnel in 2014 and in the field in 2015 and 2016 are in accordance with the expected results, with K3 being the most resistant genotype followed by I2 and Boléro, and H1 as the most susceptible.

A PCA confirmed the difference between the resistant genotypes (I2, K3 and Boléro) and H1, the susceptible one (Figs 1 and 2). These differences were not characterized by absence or presence of metabolites (qualitative difference), except for cis-α-bergamotene, which was only accumulated by H1, but by a different level of accumulation of each metabolite among the four genotypes (i.e. quantitative differences). As the differences are quantitative and not qualitative, if the metabolites concerned are ever linked to resistance, this would be consistent with the partial nature of carrot resistance to *A. dauci* described by Strandberg *et al*.^40^ or Le Clerc *et al*.^20,21^ and the fact that, up to now, no major resistance gene has been found.

Among the seven terpenes that differentiate resistant genotypes from H1 (Table 3), the γ-terpinene effectively differentiated all genotypes from others, but H1 was ranked between Boléro and I2. Therefore, this metabolite is probably genotype dependent and has no link to resistance status. Among the six others, three are monoterpenes, which were more accumulated by resistant genotypes, and three are sesquiterpenes, which were more accumulated by the susceptible one. We would have been able to hypothesize that the secondary metabolite flux is monitored differently between resistant and susceptible genotypes and that in this case there could be a balance between monoterpenes and sesquiterpenes. However, this seems somewhat unlikely as precursors and biosynthesis location in the cell differ between mono- and sesquiterpenes. Moreover, other sesquiterpenes such as α-bisabolene, to name but one, are highly accumulated by resistant genotypes K3 and I2 in comparison to H1 (Table S3) whereas, on the contrary, some monoterpenes, for example α-terpinolene, are more accumulated by H1 than by resistant genotypes. The difference in the patterns of the volatile organic compounds between resistant and susceptible genotypes should depend on genetic factors impacting terpene synthase family as suggested by Keilwagen *et al*.^34^. It could also originated downstream in terpene biosynthesis pathway, where the enzyme is specific to each compound e.g. α-pinene synthase EC 4.2.3.119 or cis-α-bergamotene synthase EC 4.2.3.54 in the KEGG database^31^. The accumulation of metabolites needs to be considered one by one, rather than at the sub-family level. The fact that limonene, cis-β-farnesene, cis-α-bergamotene and trans-β-farnesene, metabolites are more accumulated by H1, could be unfavorable for resistance. On the contrary, accumulation of camphene, α-pinene, α-humulene could favor resistance.

Concerning flavonoid, a thorough analysis led us to identify several compounds of putative interest in the context of Carrot-*A. dauci* relationship (Tables 4 and 5). Six compounds distinguished H1 from all the resistant genotypes (Table 6). Their biosynthesis pathway (Fig. 3) revealed that from apigenin or luteolin substrates, the activity of a 4′-glucosyltransferase leading respectively to apigenin 4′-*O*-glucoside or luteolin 4′-*O*-glucoside appears to favor resistance (as the resulting compounds are more accumulated by resistant genotypes than by H1). A similar conclusion can be drawn concerning the accumulation of apigenin 7*-O-*rutinoside obtained from apigenin 7*-O-*glucoside thanks to rhamnosyl transferase EC 2.4.1.236 activity. No studies mentioning for a link between the accumulation of these metabolites and resistance to diseases were found. However, some of these metabolites are cited in the literature in relation to carrot resistance to pests, as reported in a paper by Leiss *et al*.^41^, stating that carrot genotypes (Ingot, Nantes and D1) resistant to thrips accumulated significantly more luteolin, β-alanine and sinapic acid than susceptible ones (orange, purple–yellow, and Paris Market) in their leaves.

Our results also show that the metabolites that differentiate Boléro, I2 and K3 from H1 are not always the same in all resistant genotypes (Tables 3 and 6). We observed a different accumulation profile in the three resistant genotypes both for terpenes and flavonoids. Certain compounds differentiate H1 from one or two resistant genotypes but not all three. Some differences in geographical origin or genetic background between the three sources of resistance could partially explain these results. As mentioned above, I2 and K3 both originate from Japan but they have morphological differences. I2 looks like the orange carrot type “Kuroda” while K3 appears to be phenotypically close to orange carrot type “Oonaga”. Boléro is a French orange carrot type “Nantaise” produced in the Vilmorin breeding program, which is less resistant than K3 and I2. Using molecular analyses with SSR markers, Le Clerc *et al*.^21^ identified differences in genetic backgrounds between H1, I2 and K3. Numerous additional data suggested different resistance mechanisms between Boléro, I2 and K3 against *A. dauci*: induction of PR4, a gene from the jasmonic acid pathway, in K3 but not in I2 after *A. dauci* inoculation^42^, I2 being more resistant to fungal extract than Boléro and K3^43^, complementary favorable alleles for different rQTLs in I2 or K3^21^. From these previous results, we hypothesize that Boléro, I2 and K3 hosted different resistant mechanisms against *A. dauci* that could originate from differences in their secondary metabolite profile. This also means that compounds that are not common to the three resistant genotypes but that at least differ between one of them and H1 may be of interest for further analysis. In that respect, α-bisabolene (also for I2), caryophyllene, α-humulene, β-bisabolene, bornyl acetate, cis-β-ocimene, α-copaene (also for Boléro) and feruloylquinic acid could also contribute to K3 resistance. Differences between resistant and susceptible genotypes could also be due to a fortuitous event with no link with resistance status, only a genotype effect.

Different accumulation patterns of chrysoeriol derivatives may be the result of a competition between a rhamnosyl transferase EC 2.4.1.236 and a malonyltransferase EC 2.3.1.115 for the chrysoeriol 7*-O-*glucoside substrate, leading respectively to accumulation of chrysoeriol 7*-O-* rutinoside and to chrysoeriol 7*-O-* malonylglucoside (Fig. 3). This balance is possibly associated to better resistance, as chrysoeriol 7*-O-*rutinoside was accumulated by resistant genotypes whereas chrysoeriol 7*-O-* malonylglucoside was more accumulated by H1. A similar pattern was observed for luteolin derivatives, luteolin 7*-O-*rutinoside being more accumulated in the resistant I2 and Boléro genotypes than in the susceptible H1.

More generally, I2 and Boléro appeared to be relatively close to each other concerning flavonoid accumulation (Fig. 2). Even if I2 is Kuroda type whereas Boléro is Nantaise type, it is possible that I2 or one of its relatives is Boléro’s ancestor. Indeed, in the last century, Kuroda type material has been widely used by European carrot breeders to enhance the resistance level of their breeding Nantaise type material.

A bulk segregant analysis with a set of resistant and susceptible progenies, originating from the cross between K3 and H1 was realized to validate or to reject the involvement of metabolite candidates in resistance. In accordance with the results obtained in the two parental lines H1 and K3, we observed that luteolin 7*-O-*glucuronide on one hand and feruloylquininc acid, camphene, α-pinene, apigenin 4′*-O-*glucoside, luteolin 4′*-O-*glucoside, caryophyllene and β-bisabolene on the other hand were differentially accumulated in the susceptible and resistant bulk (Table 7). This result confirms our hypothesis that higher accumulation of these metabolites is associated with the resistance level of K3 and could be inherited across generations. Conversely, α-bisabolene, bornyl acetate, α-humulene, Z-β-ocimene, and limonene, cis-α-bergamotene, cis-β-farnesene, luteolin 7*-O-*glucoside, trans-β-farnesene, chrysoeriol 7*-O-*malonylglucoside, which also differentiated K3 from H1, are probably not directly linked to K3 resistance, as they were not differentially accumulated in the susceptible and the resistant bulk (Table 7).

Even if a higher accumulation of feruloylquininc acid, camphene, α-pinene, apigenin 4′*-O-*glucoside, luteolin 4′*-O-*glucoside, caryophyllene and β-bisabolene, participate in the partial resistance of K3, nothing is known about a putative role of this set of secondary metabolites and luteolin 7*-O-*glucuronide in the interaction between carrot and *A. dauci*. However, different studies have reported that some of these metabolites have antifungal activities in other species. For example, Bily *et al*.^18^ demonstrated antifungal activity of dehydromers of ferulic acid (DFA) against *Fusarium graminarium*, which is responsible for Gibberella ear rot (GEA) of maize. DFA inhibited mycelium propagation by strengthening the maize grain cell wall. feruloylquinic acid is also a phenolic acid, and could thus act like DFA against *A. dauci*. Džamić *et al*.^44^ showed that deodorized aqueous extract, with feruloylquininc acid as one principal component, from *Hyssopus officinalis* had an antifungal activity *in vitro* against two and four *Cladosporium* and *Aspergillus* species, respectively. α-pinene (a component of tea tree oil) displayed *in vitro* antifungal activity against *Aspergillus niger* and *A. flavus* by inhibiting the proliferation of their mycelia^9^. A vapor treatment of lemon leaves with α-pinene was also able to induce the expression of the allene oxide synthase gene, a defense-related gene (Yamasaki *et al*.)^45^. Alexa *et al*.^46^ showed that essential oil from *Salvia officinalis*, the main compounds of which are caryophyllene, camphene and β-pinene, had a negative effect on proliferation of *Fusarium graminearum in vitro*. Finally, Vitalini *et al*.^47^ showed that luteolin-4′*-O-*glucoside has a moderate effect against *Plasmodium falciparum*, and, in a very recent study, Lin *et al*.^48^, concluded that luteolin and luteolin-4′*-O-*glucoside could be developed as therapeutics for human diseases. To our knowledge, no biological activity has been reported for apigenin 4′*-O-*glucoside. From the literature mentioned above, we can hypothesize that secondary metabolites could also be favorable for carrot partial resistance to *A. dauci* and are good candidates to explain resistance mechanisms. If confirmed, they therefore could also be good targets for resistance breeding programs.

To conclude, interconnection between secondary metabolite pathways could probably explain the complexity of the observed results. Indeed, one compound could be more important for resistance than another but because the secondary metabolism pathway is all interconnected, increasing this particular compound might influence several intermediate compounds or several other final compounds. Simlat *et al*.^49^ also pinpointed that ratios between phenolic compounds were higher for the carrot lines that were resistant to carrot fly, confirming that metabolites could not probably be considered alone to improve resistance. As metabolomic analyses are not exhaustive and because we worked on a small set of genotypes, it is not excluded that some of the non-differentially accumulated metabolites could be differential with another set of resistant genotypes. However, as we worked on different sources of resistance, we probably identified a significant part of the metabolites involved in carrot resistance to *A. dauci*. Differential metabolites selected in this work, which are not identified yet will be further characterized by detailed analysis of their mass spectra. Further analyses will be aimed at characterizing the involvement and the role of those secondary metabolites in resistance against *A. dauci* and possibly to link them to rQTLs previously identified^21^. In the absence of isogenic mutants or pure lines in carrot, a study of segregating populations for resistance is potentially of interest. To this end, a metabolite-QTL (mQTL) analysis after *A. dauci* attack in field using the same two segregating populations obtained from H1-I2 and H1-K3 hybridizations used for rQTL detection is currently underway to identify co-localization between mQTL and rQTL. A microarray analysis is also planned to characterize gene expression in the three genotypes, H1, K3 and I2 after inoculation with *A. dauci*. The integrated analysis of metabolome and transcriptome data should provide good candidates for further functional validation with transformation.

## Electronic supplementary material

Supplementary information

## References

[1] Wink M (1988). Plant breeding: importance of plant secondary metabolites for protection against pathogens and herbivores. Theor. Appl. Genet..

[2] Kliebenstein DJ (2004). Secondary metabolites and plant/environment interactions: a view through Arabidopsis thaliana tinged glasses. Plant, Cell Environ..

[3] Dudareva N, Negre F, Nagegowda DA, Orlova I (2006). Plant volatiles: recent advances and future perspectives. CRC. Crit. Rev. Plant Sci..

[4] Kroymann J (2011). Natural diversity and adaptation in plant secondary metabolism. Curr. Opin. Plant Biol..

[5] Sade D (2015). Comparative metabolomics and transcriptomics of plant response to Tomato yellow leaf curl virus infection in resistant and susceptible tomato cultivars. Metabolomics.

[6] Cuperlovic-Culf M (2016). Metabolic biomarker panels of response to fusarium head blight infection in different wheat varieties. PLoS One.

[7] Johnson CH, Ivanisevic J, Siuzdak G (2016). Metabolomics: Beyond biomarkers and towards mechanisms. Nat. Rev. Mol. Cell Biol..

[8] Hong, J., Yang, L., Zhang, D. & Shi, J. Plant metabolomics: An indispensable system biology tool for plant science. *Int. J. Mol. Sci*. **17** (2016). 10.3390/ijms17060767 PMC4926328 27258266

[9] Hammer KA, Carson CF, Riley TV (2003). Antifungal activity of the components of Melaleuca alternifolia (tea tree) oil. J. Appl. Microbiol..

[10] Shiojiri K (2006). Changing green leaf volatile biosynthesis in plants: an approach for improving plant resistance against both herbivores and pathogens. Proc. Natl. Acad. Sci. USA.

[11] Yamani HA, Pang EC, Mantri N, Deighton MA (2016). Antimicrobial Activity of Tulsi (Ocimum tenuiflorum) Essential Oil and Their Major Constituents against Three Species of Bacteria. Front. Microbiol..

[12] Dongbo L (2016). Antibacterial, antifungal and *in vitro* cytotoxic activities of three extracts isolated from mint. J. Med. Plants Res..

[13] Sharma A, Rajendran S, Srivastava A, Sharma S, Kundu B (2017). Antifungal activities of selected essential oils against Fusarium oxysporum f. sp. lycopersici 1322, with emphasis on Syzygium aromaticum essential oil. J. Biosci. Bioeng..

[14] Singh B, Sharma RA (2015). Plant terpenes: defense responses, phylogenetic analysis, regulation and clinical applications. 3 Biotech.

[15] Prisic S, Xu M, Wilderman PR, Peters RJ (2004). Rice Contains Two Disparate ent-Copalyl Diphosphate Synthases with Distinct Metabolic Functions. PLANT Physiol..

[16] Rodriguez A (2014). Terpene Down-Regulation Triggers Defense Responses in Transgenic Orange Leading to Resistance against Fungal Pathogens. PLANT Physiol..

[17] Mace ME, Bell AA, Stipanovic RD (1978). Histochemistry and identification of flavanols in Verticillium wilt-resistant and -susceptible cottons. Physiol. Plant Pathol..

[18] Bily AC (2003). Dehydrodimers of Ferulic Acid in Maize Grain Pericarp and Aleurone: Resistance Factors to Fusarium graminearum. Phytopathology.

[19] Farrar JJ, Pryor BM, Davis RM (2004). Alternaria diseases of carrot. Plant Dis..

[20] Le Clerc V, Pawelec A, Birolleau-Touchard C, Suel A (2009). & Briard, M. Genetic architecture of factors underlying partial resistance to Alternaria leaf blight in carrot. Theor. Appl. Genet..

[21] Le Clerc V (2015). QTL mapping of carrot resistance to leaf blight with connected populations: stability across years and consequences for breeding. Theor. Appl. Genet..

[22] Lecomte M (2012). Inhibitory effects of the carrot metabolites 6-methoxymellein and falcarindiol on development of the fungal leaf blight pathogen Alternaria dauci. Physiol. Mol. Plant Pathol..

[23] Benichou S, Dongo A, Henni DE, Peltier D, Simoneau P (2009). Isolation and characterization of microsatellite markers from the phytopathogenic fungus Alternaria dauci. Mol. Ecol. Resour..

[24] Pawelec A, Dubourg C, Briard M (2006). Evaluation of carrot resistance to alternaria leaf blight in controlled environments. Plant Pathol..

[25] Boedo C (2012). Evaluating aggressiveness and host range of Alternaria dauci in a controlled environment. Plant Pathol..

[26] Smith CA (2006). XCMS: processing mass spectrometry data for metabolite profiling using Nonlinear Peak Alignment,Matching,and Identification. ACS Publ..

[27] van den Berg RA, Hoefsloot HCJ, Westerhuis JA, Smilde AK, van der Werf MJ (2006). Centering, scaling, and transformations: improving the biological information content of metabolomics data. BMC Genomics.

[28] Smith, C. *et al*. METLIN: A metabolite mass spectral database. Therapeutic drug monitoring **27** (2005). 10.1097/01.ftd.0000179845.53213.39 16404815

[29] Feeny P, Sachdev K, Rosenberry L, Carter M (1988). Luteolin 7-O-(6″-O-malonyl)-β-d-glucoside and trans-chlorogenic acid: Oviposition stimulants for the black swallowtail butterfly. Phytochemistry.

[30] Teubert H, Wunscher G, Herrmann K (1977). Flavonole und Flavone der Gemüsearten. Z. Lebensm. Unters. Forsch..

[31] Kanehisa M, Furumichi M, Tanabe M, Sato Y, Morishima K (2017). KEGG: New perspectives on genomes, pathways, diseases and drugs. Nucleic Acids Res..

[32] Kainulainen P, Tarhanen J, Tiilikkala K, Holopainen JK (1998). Foliar and Emission Composition of Essential Oil in Two Carrot Varieties. J. Agric. Food Chem..

[33] Ulrich D, Nothnagel T, Schulz H (2015). Influence of Cultivar and Harvest Year on the Volatile Profiles of Leaves and Roots of Carrots (Daucus carota spp. sativus Hoffm.). J. Agric. Food Chem..

[34] Keilwagen J (2017). The Terpene Synthase Gene Family of Carrot (Daucus carota L.): Identification of QTLs and Candidate Genes Associated with Terpenoid Volatile Compounds. Front. Plant Sci..

[35] Verma N, Shukla S (2015). Impact of various factors responsible for fluctuation in plant secondary metabolites. J. Appl. Res. Med. Aromat. Plants.

[36] Nithia SMJ, Shanthi N, Kulandaivelu G (2005). Different responses to UV-B enhanced solar radiation in radish and carrot. Photosynthetica.

[37] Ibrahim MA, Nissinen A, Prozherina N, Oksanen EJ, Holopainen JK (2006). The influence of exogenous monoterpene treatment and elevated temperature on growth, physiology, chemical content and headspace volatiles of two carrot cultivars (Daucus carota L.). Environ. Exp. Bot..

[38] Ceoldo S (2009). Metabolomics of Daucus carota cultured cell lines under stressing conditions reveals interactions between phenolic compounds. Plant Sci..

[39] Seljåsen R (2012). Effects of genotype, soil type, year and fertilisation on sensory and morphological attributes of carrots (Daucus carota L.). J. Sci. Food Agric..

[40] Strandberg JO, Bassett MJ, Peterson CE, Berger RD (1972). Sources of resistance to Alternaria dauci. in. HortScience.

[41] Leiss KA, Cristofori G, van Steenis R, Verpoorte R, Klinkhamer PGL (2013). An eco-metabolomic study of host plant resistance to Western flower thrips in cultivated, biofortified and wild carrots. Phytochemistry.

[42] Lecomte, M. Thèse Mickaël LECOMTE. 1–183 (2013).

[43] Lecomte M (2014). Partial Resistance of Carrot to Alternaria dauci Correlates with *In Vitro* Cultured Carrot Cell Resistance to Fungal Exudates. PLoS One.

[44] Džamić AM (2013). Composition, antifungal and antioxidant properties of Hyssopus officinalis L. subsp. pilifer (Pant.) Murb. essential oil and deodorized extracts. Ind. Crops Prod..

[45] Yamasaki Y (2007). Biological roles of monoterpene volatiles derived from rough lemon (Citrus jambhiri Lush) in citrus defense. J. Gen. Plant Pathol..

[46] Alexa E (2018). Synergistic Antifungal, Allelopatic and Anti-Proliferative Potential of Salvia officinalis L., and Thymus vulgaris L. Essential Oils. Molecules.

[47] Vitalini S (2011). Phenolic compounds from Achillea millefolium L. and their bioactivity. Acta Biochim. Pol..

[48] Lin Y (2018). Luteolin-4′-O-glucoside and its aglycone, two major flavones of Gnaphalium affine D. Don, resist hyperuricemia and acute gouty arthritis activity in animal models. Phytomedicine.

[49] Simlat M, Stobiecki M, Szklarczyk M (2013). Accumulation of selected phenolics and expression of PAL genes in carrots differing in their susceptibility to carrot fly (Psila rosae F.). Euphytica.

