# Supplementary material for: Link between carrot leaf secondary metabolites and resistance to Alternaria dauci
Source: Sci Rep. 2018 Sep 13;8:13746. doi: 10.1038/s41598-018-31700-2 (PMC6137067; doi:10.1038/s41598-018-31700-2)
Supplement: Supplementary file 1 — Supplementary information [file 41598_2018_31700_MOESM1_ESM.pdf]

Link between carrot leaf secondary metabolites and resistance to *Alternaria dauci*

Claude Koutouan, Valérie Le Clerc*, Raymonde Baltenweck*, Patricia Claudel, David Halter, Philippe Hugueney, Latifa Hamama, Anita Suel, Sébastien Huet, Marie-Hélène Bouvet Merlet & Mathilde Briard

*valerie.leclerc@agrocampus-ouest.fr

*raymonde.baltenweck@inra.fr

Supplementary information

Supplementary Table S1 Evaluation of resistance to *Alternaria dauci*

Parental lines and cultivars	Mean disease index * of a 10 year evaluation
H1	7
Boléro	4.3
I2	3.8
K3	3.4

F3 segregating progenies	Mean disease index * of a 3 year evaluation
PC3-265	3.8
PC3-241	4
PC3-109	4.2
PC3-196	4.3
PC3-222	4.3
PC3-176	4.5
PC3-226	4.5
PC3-249	4.5
PC3-263	4.5
PC3-40	4.6
PC3-105	6.3
PC3-135	6.3
PC3-154	6.3
PC3-4	6.3
PC3-78	6.5
PC3-106	6.7
PC3-83	6.7
PC3-240	6.8
PC3-262	6.8
PC3-12	6.9

* Disease index obtained as described by Le Clerc *et al.* (2009) based on a 0 to 9 scale (0 = no visible disease damage on leaves, 9 = leaves totally blighted)
PC3 is a segregating population obtained from a cross between H1 and K3 genotypes

a

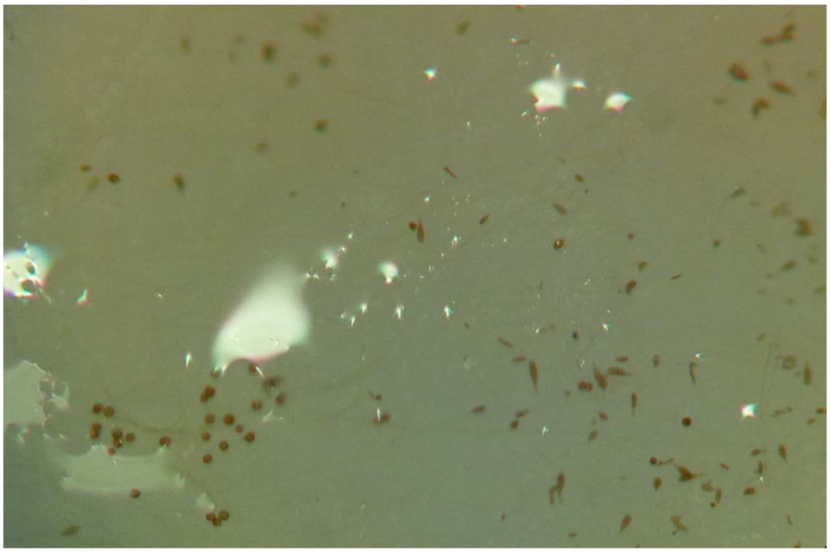

b

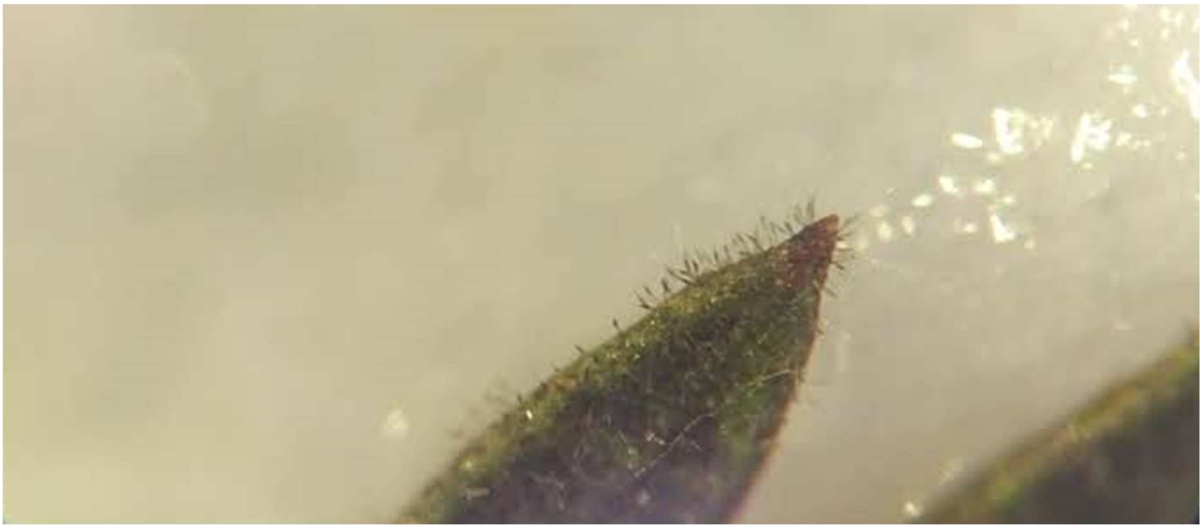

Supplementary Figure S1 Isolation of *Alernaria dauci* from carrot leaves in Blagon and Ychoux Field after attack prediction. (a) Blagon 2015: Observation of conidies on binocular loup NIKON SMZ 1000 plan Apo - 1X - WD70 / Agrandissement 6X. (b) Ychoux 2016: Observation of conidies on binocular loup NIKON SMZ 1000 plan Apo - 1X - WD70 / Agrandissement 4X

Supplementary Table S2 Secondary metabolites highlighted by profiling. Full name and subfamily of known metabolites are given

**Ion n° (LCMS)**	**Full name**	**Sub-Family**
5	Chlorogenic_acid	chlorogenic acid
10	Feruloylquinic_acid	chlorogenic acid
11	Luteolin 7-*O*-rutinoside	Flavone
15	Luteolin 7-*O*-glucoside	Flavone
21	Apigenin 7-*O*-rutinoside	Flavone
25	Luteolin 7-*O*-glucuronide	Flavone
27	chrysoeriol 7-*O*-rutinoside	Flavone
35	Apigenin 7-*O*-glucoside	Flavone
36	Luteolin 4'-*O*-glucoside	Flavone
39	Apigenin 4'-*O*-glucoside	Flavone
42	Chrysoeriol 7-*O*-glucoside	Flavone
44	Chrysoeriol 7-*O*-glucuronide	Flavone
45	M611T367	unknown
47	Apigenin 7-*O*-malonyl-glucoside	Flavone
48	Luteolin 7-*O*-malonyl-glucoside	Flavone
50	Chrysoeriol 7-*O*-malonyl-glucoside	Flavone

**Code**	**Full name**	**Sub-Family**
aAmo	α-Amorphene	Sesquiterpene
aBis	α-Bisabolene	Sesquiterpene
aCop	α-Copaene	Sesquiterpene
aHum	α-Humulene	Sesquiterpene
aPi	α-Pinene	Monoterpene
aTer	α-Terpinolene	Monoterpene
Bac	Bornyl acetate	Monoterpene
bBis	β-Bisabolene	Sesquiterpene
bCub	β-Cubebene	Sesquiterpene
bMyr	β-Myrcene	Monoterpene
bPhe	β-Phellandrene	Monoterpene
bPi	β-Pinene	Monoterpene
bSel	β-Selinene	Sesquiterpene
caBer	cis-α-Bergamotene	Sesquiterpene
Camp	Camphene	Monoterpene
Cary	Caryophyllene	Sesquiterpene
cbFar	cis-β-Farnesene	Sesquiterpene
cRo	cis-Rose oxide	Monoterpene
dCad	δ-Cadinene	Sesquiterpene
EaFar	cis-α-Farnesene	Sesquiterpene
EbOc	cis-β-Ocimene	Monoterpene
GerD	Germacrene D	Sesquiterpene
gTer	γ-Terpinene	Monoterpene
Lim	Limonene	Monoterpene
Lin	Linalol	Monoterpene
pCy	p-Cymene	Monoterpene
Sab	Sabinene	Monoterpene
taBer	trans-α-Bergamotene	Sesquiterpene
tbFar	trans-β-Farnesene	Sesquiterpene
ZbOc	trans-β-Ocimene	Monoterpene

Supplementary Table S3 Integrated peak of selected metabolites in four genotypes of carrot over three years

	2014-H1	2014-Boléro	2014-I2	2014-K3	2015-H1	2015-Boléro	2015-I2	2015-K3	2016-H1	2016-Boléro	2016-I2	2016-K3
chlorogenic_acid	99670337	98874432	78147831	117936659	67723644	23301823	50413172	66996796	27461093	31181390	30993571	48214646
feruloylquinic_acid	2155653	2988953	2352024	5742113	673365	642683	707434	1815203	367806	644346	549541	1350678
luteolin 7-O-rutinoside	5712791	13909266	14576633	3326505	1071346	17049779	13735467	1631092	838863	7449632	12479541	790380
luteolin 7-O-glucoside	145247684	61317611	72425104	61746587	91195646	71574558	73097571	73284296	58745238	41103197	50223857	33031273
luteolin 7-O-glucuronide	33498058	23649734	8428027	1778686	50463978	43104983	40722094	11393821	33495658	26931513	27766822	3860882
apigenin 7-O-rutinoside	10846273	17848265	46356772	17854170	1047318	8273596	10743815	7678015	1705583	3775559	14186326	5732825
chrysoeriol 7-O-rutinoside	5333263	13558807	24334383	2551273	467793	14080679	11111681	1075291	554107	5477313	10700462	689063
apigenin 7-O-glucoside	132133852	20629342	52471644	116426596	51418951	11455239	21937418	85582729	47397339	7053278	17849271	40257586
luteolin 4'-O-glucoside	11491083	47461722	13788586	20775926	303793	27154125	7844941	14524121	919922	21522889	8154416	10184366
apigenin 4'-O-glucoside	6147740	20876207	7433186	28888658	26043	4670534	1875678	7895570	462271	4749051	3777039	8306573
chrysoeriol 7-O-glucoside	96655169	28228623	43519612	50054052	26385958	19599150	19501153	29067782	24533244	11538071	14286405	17336479
luteolin 7-O-malonyl-glucoside	3986065	2704174	1155109	2774314	21460159	16184076	15351275	23594187	3392072	3521973	3311371	4426826
chrysoeriol 7-O-glucuronide	7644597	7054663	3114191	605672	6089880	9127316	6953389	1911339	3823016	4023987	4367617	650643
M611T367	15090486	1996924	885403	33614	3000173	1292440	440970	5659	2747601	1919402	917866	3455
apigenin 7-O-malonyl-glucoside	7956895	2197511	2270347	7179392	16758808	4041887	5234701	17331279	4642517	709461	1649027	3537392
chrysoeriol 7-O-malonyl-glucoside	7904692	3306821	1366260	2933142	11633646	7294162	3198398	7037858	3128337	1478467	880449	1747710
α-Amorphene	0.1449	0.0000	0.0798	0.1383	0.5811	0.0757	0.5493	0.9772	0.1792	0.0000	0.2418	0.3130
α-Bisabolene	0.0247	0.2087	0.4525	0.3344	0.1434	0.5154	1.9025	4.3103	0.1550	0.1609	0.3574	0.3621
α-Copaene	0.0418	0.0000	0.0404	0.0796	0.1556	0.0000	0.1932	0.2923	0.0508	0.0000	0.0924	0.1390
α-Humulene	1.0343	0.8873	0.7477	1.6235	1.5607	1.7729	1.5121	3.7509	0.7742	1.4543	1.1544	1.9764
α-Pinene	25.2219	32.8694	28.7285	32.6487	3.5545	21.2970	10.7548	15.4426	5.8779	11.7662	24.1373	17.5144
α-Terpinolene	1.1435	0.5496	0.4630	1.0333	5.2006	3.0172	2.1446	5.8641	4.1175	2.8919	2.3535	2.2282
Bornyl acetate	0.2163	0.2400	0.0000	0.6762	0.1093	0.1349	0.0000	0.6842	0.0000	0.0000	0.0000	0.0539
β-Bisabolene	0.0426	0.0000	0.1059	0.2251	0.0000	0.0593	0.4767	1.9446	0.0000	0.0000	0.0000	0.0335
β-Cubebene	0.1632	0.0000	0.0455	0.1452	0.0000	0.1744	0.0000	0.1515	0.2709	0.0000	0.3491	0.4496
β-Myrcene	80.8245	52.5833	60.5321	33.1577	82.5291	45.6545	94.9378	43.9561	42.3006	56.5664	82.9736	17.2313
β-Phellandrene	3.3183	2.4005	2.5838	2.3385	3.2582	2.6881	3.4982	2.2738	1.6336	3.3209	3.6988	1.1155
β-Pinene	2.7448	3.8537	2.4440	3.6243	1.5255	2.0601	1.5687	2.1990	2.3209	2.6271	2.4209	2.1659
β-Selinene	1.7069	0.2093	1.2555	1.5113	0.2040	1.1207	0.7404	0.2605	2.3114	0.4081	1.2305	0.1224
cis-α-Bergamotene	0.2249	0.0000	0.0000	0.0000	1.1146	0.0000	0.0000	0.0000	0.1539	0.0000	0.0000	0.0000
Camphene	1.3086	1.9287	1.5431	1.6939	0.1224	0.9523	0.4047	0.5497	0.2835	1.0234	1.0743	1.1018
Caryophyllene	3.1039	1.8942	2.6854	4.2850	5.9757	7.8815	6.4084	11.3222	2.5492	3.4495	4.5424	6.0688
cis-β-Farnesene	0.8675	0.0295	0.0000	0.0000	2.0790	0.1818	0.0000	0.0000	0.6772	0.0812	0.0000	0.0000
cis-Rose oxide	0.1027	0.0000	0.1026	0.1506	5.4699	0.1319	5.4398	5.6007	0.1400	0.1298	0.1424	0.1342
δ-Cadinene	0.5516	0.0910	0.3596	0.4273	1.9575	0.3594	1.7612	3.0993	0.5827	0.1777	0.7122	0.9514
cis-α-Farnesene	0.2007	0.0000	0.3554	0.6812	0.4819	0.2063	1.1670	2.9352	0.1554	0.1412	0.7184	1.1228
cis-β-Ocimene	2.4517	2.1913	1.3148	1.8637	14.6266	6.3186	14.1512	10.3272	2.3743	3.7688	4.0237	2.4038
Germacrene D	2.9952	0.7964	2.2008	2.6470	4.0308	2.2806	3.0893	6.8120	4.3831	1.0118	5.5708	7.3043
γ-Terpinene	3.2356	2.7119	1.9589	4.8952	5.0428	2.3076	1.5980	7.2908	2.6270	2.3459	1.2234	3.2675
Limonene	20.6659	8.1704	7.4895	6.6725	9.5324	6.5808	6.5547	5.9714	6.5311	5.3580	5.6317	2.7008
Linalol	0.0000	0.0000	0.2043	0.0000	0.3811	0.0331	0.2661	0.4645	0.0000	0.0000	0.1220	0.0000
p-Cymene	2.4035	2.3957	2.0775	2.3138	5.2682	5.3653	4.3989	6.2113	2.3261	3.3454	1.6523	2.6046
Sabinene	62.9637	79.9607	41.0824	124.3401	45.0915	30.8917	7.8744	53.1481	79.1255	38.1565	22.4251	73.9480
trans-α-Bergamotene	0.6632	0.7576	0.0286	0.0142	1.4609	1.3603	0.0667	0.1373	0.5165	0.7788	1.7365	2.3241
trans-β-Farnesene	0.1536	0.0000	0.0000	0.0000	0.3689	0.0000	0.0000	0.0000	0.1163	0.0000	0.0000	0.0000
trans-β-Ocimene	1.0994	1.1263	0.2601	5.3855	5.5643	1.8717	2.2131	27.5904	0.7002	0.9400	0.4749	5.1390

Supplementary Table S4 Mean of autoscaled data per year per genotype. Each year corresponds to one environment. These data were used for environment and genotype effect analyses, PCA, Heatmap, SNK test.

Genotype	2014-Boléro	2015-Boléro	2016-Boléro	2014-I2	2015-I2	2016-I2	2014-K3	2015-K3	2016-K3	2014-H1	2015-H1	2016-H1
Environment	Angers	Blagon	Ychoux	Angers	Blagon	Ychoux	Angers	Blagon	Ychoux	Angers	Blagon	Ychoux
Group	Boléro	Boléro	Boléro	I2	I2	I2	K3	K3	K3	H1	H1	H1
Label	Resistant	Resistant	Resistant	Resistant	Resistant	Resistant	Resistant	Resistant	Resistant	Susceptible	Susceptible	Susceptible
chlorogenic_acid	0.01	-1.21	-0.26	-1.20	-0.07	-0.12	1.13	0.63	1.10	0.06	0.66	-0.56
feruloylquinic_acid	-0.21	-0.61	-0.20	-0.64	-0.48	-0.32	1.62	1.64	1.47	-0.77	-0.55	-0.85
luteolin 7-*O*-rutinoside	0.75	1.16	0.37	0.86	0.71	1.02	-1.00	-0.90	-0.82	-0.60	-0.97	-0.81
luteolin 7-*O*-glucoside	-0.64	-0.63	-0.40	-0.34	-0.46	0.64	-0.63	-0.44	-1.08	1.61	1.52	1.10
luteolin 7-*O*-glucuronide	0.51	0.43	0.32	-0.62	0.28	0.58	-1.12	-1.60	-1.56	1.24	0.90	0.85
apigenin 7-*O*-rutinoside	-0.26	0.29	-0.43	1.12	0.83	0.92	-0.26	0.16	-0.10	-0.60	-1.28	-0.78
chrysoeriol 7-*O*-rutinoside	0.21	1.16	0.24	1.27	0.69	1.09	-0.88	-0.88	-0.78	-0.60	-0.98	-0.81
apigenin 7-*O*-glucoside	-1.20	-1.07	-1.20	-0.56	-0.71	-0.44	0.73	1.48	0.69	1.04	0.30	1.09
luteolin 4'-*O*-glucoside	1.53	1.43	1.35	-0.61	-0.45	-0.25	-0.17	0.20	0.00	-0.76	-1.18	-1.11
apigenin 4'-*O*-glucoside	0.44	0.35	0.14	-0.74	-0.58	-0.10	1.15	1.42	1.27	-0.85	-1.19	-1.23
chrysoeriol 7-*O*-glucoside	-0.95	-0.85	-0.99	-0.40	-0.87	-0.18	-0.16	1.15	0.08	1.51	0.58	1.40
luteolin 7-*O*-malonyl-glucoside	0.04	-0.66	-0.14	-1.32	-0.84	-0.04	0.10	0.99	0.74	1.17	0.51	-0.26
chrysoeriol 7-*O*-glucuronide	0.70	1.10	0.48	-0.42	0.33	1.03	-1.14	-1.45	-1.53	0.87	0.02	0.36
M611T367	-0.41	0.09	0.46	-0.59	-0.64	-0.23	-0.73	-1.02	-1.24	1.72	1.57	1.20
apigenin 7-*O*-malonyl-glucoside	-0.91	-1.04	-1.10	-0.88	-0.86	-0.36	0.76	1.00	0.52	1.02	0.91	1.15
chrysoeriol 7-*O*-malonyl-glucoside	-0.22	0.00	-0.36	-0.95	-1.35	-0.90	-0.36	-0.08	-0.07	1.52	1.43	1.43
α-Amorphene	-1.35	-1.27	-1.37	-0.16	0.01	0.44	0.71	1.17	0.97	0.81	0.1	-0.03
α-Bisabolene	-0.25	-0.64	-0.84	1.08	0.1	0.85	0.43	1.37	0.89	-1.26	-0.83	-0.89
α-Copaene	-1.24	-1.32	-1.19	0	0.27	0.37	1.2	1.09	1.16	0.04	-0.04	-0.33
α-Humulene	-0.48	-0.35	0.23	-0.85	-0.59	-0.37	1.43	1.49	1.25	-0.1	-0.55	-1.11
α-Pinene	0.83	1.14	-0.39	-0.31	-0.27	1.19	0.77	0.36	0.34	-1.28	-1.23	-1.14
α-Terpinolene	-0.73	-0.59	-0.01	-0.98	-1.09	-0.63	0.69	1.03	-0.78	1.02	0.65	1.41
Bornyl acetate	-0.15	-0.32	-0.5	-1	-0.76	-0.5	1.39	1.47	1.5	-0.24	-0.4	-0.5
β-Bisabolene	-0.95	-0.62	-0.5	0.13	-0.16	-0.5	1.34	1.46	1.5	-0.52	-0.68	-0.5
β-Cubebene	-1.13	0.98	-1.39	-0.55	-0.86	0.42	0.72	0.74	0.95	0.95	-0.86	0.02
β-Myrcene	-0.21	-0.82	0.25	0.19	1.09	1.21	-1.2	-0.88	-1.18	1.22	0.61	-0.27
β-Phellandrene	-0.58	-0.44	0.7	-0.17	1.03	1	-0.71	-1.18	-1.05	1.46	0.59	-0.64
β-Pinene	1.01	0.65	1.26	-1.06	-0.79	0.19	0.67	1.06	-1.13	-0.62	-0.92	-0.32
β-Selinene	-1.44	1.25	-0.62	0.13	0.37	0.22	0.51	-0.74	-0.91	0.8	-0.87	1.32
cis-α-Bergamotene	-0.5	-0.5	-0.5	-0.5	-0.5	-0.5	-0.5	-0.5	-0.5	1.5	1.5	1.5
Camphene	1.19	1.29	0.39	-0.29	-0.3	0.52	0.29	0.12	0.59	-1.19	-1.11	-1.49
Caryophyllene	-1.1	-0.01	-0.46	-0.31	-0.61	0.26	1.3	1.41	1.26	0.11	-0.79	-1.06
cis-β-Farnesene	-0.45	-0.38	-0.33	-0.52	-0.56	-0.58	-0.52	-0.56	-0.58	1.5	1.49	1.49
cis-Rose oxide	-1.4	-1.5	-1.19	0.22	0.48	1.02	0.97	0.54	-0.43	0.22	0.49	0.6
δ-Cadinene	-1.37	-1.28	-1.32	0.01	-0.03	0.33	0.36	1.16	1.07	1	0.15	-0.07
cis-α-Farnesene	-0.84	-0.59	-0.67	-0.40	0.60	0.05	0.00	2.80	0.55	-0.60	-0.25	-0.65
cis-β-Ocimene	-0.68	0.17	-0.35	-0.86	1.79	-0.30	-0.75	1.00	-0.64	-0.63	1.89	-0.64
Germacrene D	-1.41	-0.9	-1.34	0.04	-0.49	0.38	0.5	1.4	1.03	0.87	-0.01	-0.07
γ-Terpinene	-0.39	-0.67	-0.02	-1	-0.94	-1.34	1.36	1.23	1.06	0.03	0.38	0.31
Limonene	-0.39	-0.36	0.18	-0.49	-0.38	0.35	-0.61	-0.74	-1.43	1.49	1.48	0.9
Linalol	-0.5	-1.35	-0.5	1.5	-0.11	1.5	-0.5	0.95	-0.5	-0.5	0.51	-0.5
p-Cymene	0.64	0.07	1.23	-1.45	-1.23	-1.18	0.11	1.22	0.17	0.7	-0.06	-0.22
Sabinene	0.08	-0.17	-0.55	-1.02	-1.33	-1.13	1.34	0.95	0.75	-0.4	0.55	0.93
trans-α-Bergamotene	0.98	0.8	-0.67	-0.84	-0.91	0.47	-0.88	-0.82	1.17	0.74	0.93	-0.98
trans-β-Farnesene	-0.5	-0.5	-0.5	-0.5	-0.5	-0.5	-0.5	-0.5	-0.5	1.5	1.5	1.5
trans-β-Ocimene	-0.36	-0.6	-0.39	-0.74	-0.58	-0.6	1.48	1.49	1.49	-0.38	-0.3	-0.5

Supplementary Table S5 List of 52 major ions, which best differentiated the four carrot genotypes from each other. Ions were selected with analysis of variance following analysis of all samples collected for the 4 genotypes over 3 years. Ions are numbered according to their retention time. For all ions, the number, identifier, *m/z*, and retention time (RT) are indicated. For tentatively identified ions, molecular formula and putative identifications are indicated. Identifications confirmed by using the corresponding standards are indicated in bold.

N°	Identifier	m/z	RT (s)	(putative) formula	(putative) identification
1	M127T111	127.039106	111.42		
2	M433T112	433.134043	111.87		
3	M325T112	325.091809	112.01		
4	M344T124	344.134012	123.51		
**5**	**M355T122**	**355.102303**	**122.48**	**$C_{16}H_{19}$ 09**	**3-caffeoylquinic acid**
6	M374T131	374.144518	131.38		
7	M345T180	345.154384	180.24		
8	M363T180	363.164886	180.22		
9	M177T194	177.054661	193.54	$C_{10}H_{9}O_{3}$	feruloyl fragment
10	M369T195	369.118032	194.58	$C_{17}H_{21}O_{9}$	feruloylquinic acid
**11**	**M595T208**	**595.165712**	**207.61**	**$C_{27}H_{31}O_{15}$**	**luteolin 7-*O*-rutinoside**
12	M596T208	596.169161	207.66	isotope of M595T208	
13	M179T217	179.070315	217.32		
14	M197T217	197.080964	217.32		
**15**	**M897T240**	**897.208276**	**239.92**	**$C_{42}H_{41}O_{22}$**	**luteolin 7-*O*-glucoside (dimer)**
16	M898T240	898.211615	239.92	isotope of M897T240	
17	M374T248	374.144528	247.59		
18	M397T255	397.149323	254.88		
19	M432T255	432.186418	254.93		
20	M163T257	163.03899	256.62		
21	M579T266	579.170835	266.45	$C_{27}H_{31}O_{14}$	apigenin 7-*O*-rutinoside
22	M580T266	580.174271	266.45	isotope of M579T213	
23	M287T268	287.054738	268.11	$C_{15}H_{11}O_{6}$	luteolin fragment
24	M465T269	465.09309	268.61	isotope of M463T272	
**25**	**M463T272**	**463.087006**	**271.66**	**$C_{21}H_{19}O_{12}$**	**luteolin 7-*O*-glucuronide**
26	M464T272	464.090517	271.64	isotope of M463T272	
27	M609T278	609.18138	277.68	$C_{28}H_{33}O_{15}$	chrysoeriol 7-*O*-rutinoside
28	M610T278	610.184774	277.89	isotope of M609T278	
29	M207T291	207.101658	290.83		
30	M245T291	245.080875	290.83		
31	M271T307	271.059908	307.35	$C_{15}H_{11}O_{5}$	apigenin fragment
32	M435T307	435.118481	307.34	isotope of M433T308	
33	M865T307	865.218214	307.35	dimere of M433T308	
34	M434T308	434.116245	307.72	isotope of M433T308	
**35**	**M433T308**	**433.112837**	**307.89**	**$C_{21}H_{21}O_{10}$**	**apigenin 7-*O*-glucoside**
**36**	**M449T312**	**449.107749**	**312.48**	**$C_{21}H_{21}O_{11}$**	**luteolin 4'-*O*-glucoside**
37	M450T312	450.111134	312.24	isotope of M449T312	
38	M287T312	287.05471	312.33	$C_{15}H_{11}O_{6}$	luteolin fragment
39	M433T320	433.112837	320.58	$C_{21}H_{21}O_{10}$	apigenin 4'-*O*-glucoside
40	M464T321	464.126831	321.06	isotope of M463T321	
41	M301T321	301.070491	321.07	$C_{16}H_{13}O_{6}$	chrysoeriol fragment
42	M463T322	463.12342	321.55	$C_{22}H_{33}O_{11}$	chrysoeriol 7-*O*-glucoside
43	M775T328	775.186707	328.27		
44	M477T353	477.102838	353.41	$C_{22}H_{21}O_{12}$	chrysoeriol 7-*O*-glucuronide
45	M611T367	611.139491	366.67		
46	M520T386	520.116803	385.74	isotope of M519T386	
47	M519T387	519.113411	386.50	$C_{24}H_{23}O_{13}$	apigenin 7-*O*-malonyl-glucoside
48	M535T389	535.108312	388.84	$C_{24}H_{23}O_{14}$	luteolin 7-*O*-malonyl-glucoside
49	M459T390	459.222514	390.18		
50	M549T391	549.123954	390.84	$C_{25}H_{25}O_{14}$	chrysoeriol 7-*O*-malonyl-glucoside
51	M595T400	595.144652	399.69		
52	M430T411	430.170813	410.64		

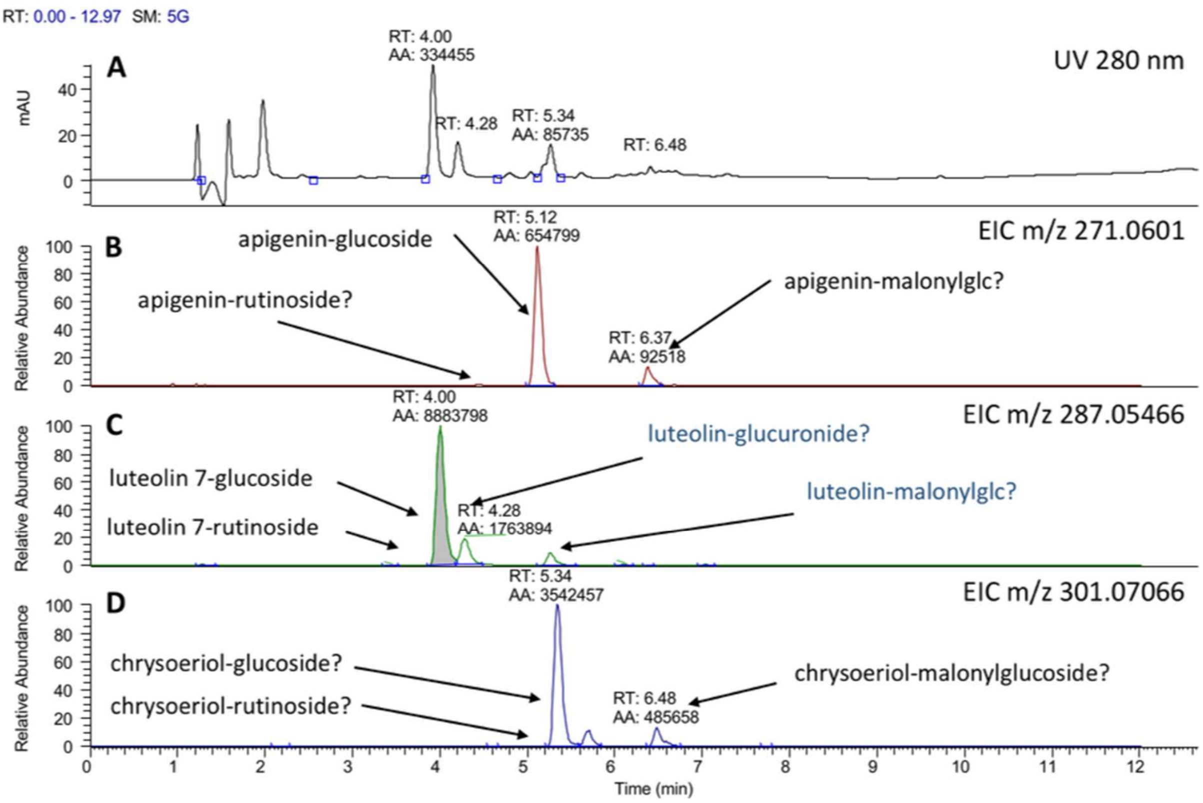

Supplementary Figure S2 UHPLC-MS analysis of major flavones in carrot leaf extract. **A**: Chromatogram with UV detection (280 nm). **B**: EIC for *m/z* 271.0601(± 2 ppm), the characteristic ion of apigenin derivatives. **C**: EIC for *m/z* 287.0546 (± 2 ppm), the characteristic ion of luteolin derivatives. **D**: EIC for *m/z* 301.0706 (± 2 ppm), the characteristic ion of chrysoeriol derivatives.

Supplementary Table S6 Identification of metabolites from carrot leaf extracts by comparison with the corresponding commercial standards. Leaf extracts and standards were compared using the same analytical conditions; *m/z* of the main ions in mass spectra and retention times are indicated.

	Standard		carrot extract	
**Full name**	RT (sec)	*m/z* of major ions	RT (sec)	*m/z* of major ions
chlorogenic acid	122	355.10234	122	355.1023
luteolin 7-O-rutinoside	208	595.16574 ; 287.05479	209	595.16571 ; 287.05474
luteolin 7-O-glucoside	240	449.10781 ; 287.05478	240	449.10775 ; 287.05474
luteolin 7-O-glucuronide	272	463.08705 ; 287.05475	272	463.08701 ; 287.05473
apigenin 7-O-glucoside	308	433.11285 ; 271.0599	308	433.11281 ; 271.05991
luteolin 4'-O-glucoside	312	449.10779 ; 287.05475	310	449.10775 ; 287.05471

Characterization of luteolin derivatives

In both the carrot leaves extracts and the luteolin 7-*O*-glucoside commercial standard, in source fragmentation produced a characteristic ion with *m/z* 287.0547 ($C_{15}H_{11}O_6$) corresponding to the flavone backbone (Supplementary Table S6). Extracted ion chromatogram (EIC) for *m/z* 287.0547 showed that some metabolites gave rise to this

particular ion in carrot leaves extracts (Supplementary Fig. S2C), indicating that these molecules may contain luteolin. For example, the ion M595T208 (n°11) with *m/z* 595.1657, corresponding to the formula $C_{27}H_{31}O_{15}$ (average mass error, AME 0.8 ppm), was putatively identified as luteolin-7-O-rutinoside. Indeed, its mass spectrum showed a fragment ion with *m/z* 287.0547 corresponding to luteolin structure. The mass difference between *m/z* 595.1657 and *m/z* 287.0547 corresponded to the loss of a neutral fragment with *m/z* 308.1111, consistent with a rutinose unit ($C_{12}H_{20}O_9$) (Supplementary Table S7). The same reasoning was applied to the ion M463T272 (n°25). The mass difference between the flavonoid fragment with *m/z* 287.0547 and the pseudo-molecular ion with *m/z* 463.0872 corresponded to the loss of a neutral fragment with *m/z* 176.0325 consistent with a glucuronide unit ($C_6H_8O_6$) (Supplementary Table S7). M463T272 was therefore putatively identified as luteolin 7-*O*-glucuronide. The same reasoning was applied to the ions M449T242, M449T310 and M535T389 (n°48), which where putatively identified as luteolin glucosides of for the two first and as luteolin malonyl-glucoside for the third. Commercial standards of luteolin 7-*O*-glucoside, luteolin 4'-*O*-glucoside, luteolin 7-*O*-rutinoside, and luteolin 7-*O*-glucuronide were therefore analyzed by UHPLC-HRMS using the same conditions as those used for carrot leaves extracts. The retention times (RTs) and fragment patterns of the standards (Supplementary Table S6) were identical to those of M897T242 (n◦15), M449T312 (n°36), M463T272 (n°25) and M595T208 (n°11) confirming their identities as luteolin 7-*O*-glucoside and luteolin 4'-*O*-glucoside, luteolin 7-*O*-rutinoside, and luteolin 7-*O*-glucuronide respectively.

Characterization of apigenin derivatives

EIC for *m/z* 271.0599 showed that several metabolites gave rise to this ion in carrot leaves extracts (Supplementary Fig. S2B), indicating that these molecules may be apigenin glycosides. This is the case for ions M433T308 (n°35) and M433T320 (n°39), with *m/z* 433.1128 corresponding to apigenin-glucosides. Their mass spectrum showed a fragment with *m/z* 271.0599 corresponding to the apigenin structure. The mass of the associated neutral fragment 162.0529 corresponded to a hexose unit to ($C_6H_{10}O_5$). The identification of M433T308 as apigenin 7-*O*-glucoside was confirmed by using the corresponding commercial standard. Based on the presence of luteolin 4'-*O*-glucoside in carrot leaf extracts, M433T320 was tentatively identified as apigenin 4'-*O*-glucoside.
The M579T268 (n°21) and M519T387 (n°47) ions also gave rise to the ion with *m/z* 271.0599 characteristic of apigenin derivatives (Supplementary Fig. S1B). Following the same reasoning they were tentatively identified as apigenin 7-*O*-rutinoside (M579T268) and apigenin 7-*O*-malonyl-glucoside (M519T387) (Supplementary Table S7).

Characterization of methyl-luteolin derivatives

EIC for *m/z* 301.0705 showed that several metabolites gave rise to this ion in carrot leaves extracts (Supplementary Fig. S2B), indicating that these molecules may be derivatives of chrysoeriol. Indeed, the mass spectra of ions M609T278, M463T322, M549T391 and M477T353 all showed the characteristic chrysoeriol ion with *m/z* 301.0705.
For each of these compounds, the mass difference between the flavonoid fragment with *m/z* 301.0705 and the pseudo-molecular ion allowed the identification of neutral fragments corresponding to different hexoses. For example, the neutral fragments with *m/z* 162.0529 ($C_6H_{10}O_5$) associated the ion M463T322 was consistent with its identification as chrysoeriol 7-

O-glucoside (3'-methyl-luteolin 7-*O*-glucoside), which has been previously reported to occur in carrot leaves (Teuber and Herrmann, 1977).
Similarly, for ions M609T278, M549T391 and M477T353, neutral fragments were tentatively identified as derived from rutinoside, malonyl-glucoside and glucuronide, respectively (Supplementary Table S7). These chrysoeriol derivatives have not been described in carrot leaves before, but their structures are consistent with those of the luteolin and apigenin derivatives identified above.

Supplementary Table S7 Analysis of mass spectra for the identification of flavones in carrot leave extracts

identifier	m/z	formula	*m/z* flavonoid fragment	formula of flavonoid fragment	flavonoid unit	mass of neutral fragment	formula	sugar unit
**M595T208**	595.1657	$C_{27}H_{31}O_{15}$	287.0547	$C_{15}H_{11}O_6$	luteolin	308.1107	$C_{12}H_{20}O_9$	rutinose
**M449T240**	449.1077	$C_{21}H_{21}O_{11}$	287.0547	$C_{15}H_{11}O_6$	luteolin	162.0529	$C_6H_{10}O_5$	glucose
**M463T272**	463.0872	$C_{21}H_{19}O_{12}$	287.0547	$C_{15}H_{11}O_6$	luteolin	176.0325	$C_6H_8O_6$	glucuronide
**M579T266**	579.17078	$C_{27}H_{31}O_{14}$	271.0599	$C_{15}H_{11}O_5$	apigenin	308.1107	$C_{12}H_{20}O_9$	rutinose
**M609T278**	609.18119	$C_{28}H_{33}O_{15}$	301.0705	$C_{16}H_{13}O_6$	chrysoeriol	308.1107	$C_{12}H_{20}O_9$	rutinose
**M433T308**	433.11274	$C_{21}H_{21}O_{10}$	271.0599	$C_{15}H_{11}O_5$	apigenin	162.0529	$C_6H_{10}O_5$	glucose
**M449T312**	449.1077	$C_{21}H_{21}O_{11}$	287.0547	$C_{15}H_{11}O_6$	luteolin	162.0529	$C_6H_{10}O_5$	glucose
**M463T322**	463.1233	$C_{22}H_{23}O_{11}$	301.0705	$C_{16}H_{13}O_6$	chrysoeriol	162.0529	$C_6H_{10}O_5$	glucose
**M535T389**	535.1081	$C_{24}H_{23}O_{14}$	287.0547	$C_{15}H_{11}O_6$	luteolin	248.0532	$C_9H_{12}O_8$	malonyl-glucose
**M519T387**	519.11332	$C_{24}H_{23}O_{13}$	271.0599	$C_{15}H_{11}O_5$	apigenin	248.0532	$C_9H_{12}O_8$	malonyl-glucose
**M549T391**	549.12377	$C_{25}H_{25}O_{14}$	301.0705	$C_{16}H_{13}O_6$	chrysoeriol	248.0532	$C_9H_{12}O_8$	malonyl-glucose
**M477T353**	477.1028	$C_{22}H_{21}O_{12}$	301.0705	$C_{16}H_{13}O_6$	chrysoeriol	176.0325	$C_6H_8O_6$	glucuronide
**M433T320**	433.1127	$C_{21}H_{21}O_{10}$	271.0599	$C_{15}H_{11}O_5$	apigenin	162.0529	$C_6H_{10}O_5$	glucose
